# Clinical Interventions and All-Cause Mortality of Patients with Chronic Kidney Disease: An Umbrella Systematic Review of Meta-Analyses

**DOI:** 10.3390/jcm9020394

**Published:** 2020-02-01

**Authors:** Jong Yeob Kim, Johanna Steingroever, Keum Hwa Lee, Jun Oh, Min Jae Choi, Jiwon Lee, Nicholas G. Larkins, Franz Schaefer, Sung Hwi Hong, Gwang Hun Jeong, Jae Il Shin, Andreas Kronbichler

**Affiliations:** 1 Yonsei University College of Medicine, Seoul 03722, Korea; crossing96@yonsei.ac.kr (J.Y.K.); ariraf2@yonsei.ac.kr (M.J.C.); 2 Department of Pediatric Nephrology, University Medical Center Hamburg-Eppendorf, Martinistraße 52, 20251 Hamburg, Germany; jo.steingroever@gmail.com (J.S.); j.oh@uke.de (J.O.); 3 Department of Pediatrics, Yonsei University College of Medicine, Yonsei-ro 50, Seodaemun-gu, C.P.O. Box 8044, Seoul 03722, Korea; AZSAGM@yuhs.ac; 4 Division of Pediatric Nephrology, Severance Children’s Hospital, Seoul 03722, Korea; 5 Department of Pediatric Nephrology, Chungnam National University Hospital, Daejeon 35015, Korea; jwmleemd@gmail.com; 6 Department of Nephrology, Perth Children’s Hospital, 15 Hospital Ave, Nedlands, WA 6909, Australia; nicholas.larkins@uwa.edu.au; 7 Centre for Kidney Research, Kids Research Institute, Westmead, NSW 2031, Australia; 8 Division of Pediatric Nephrology, Center for Pediatrics and Adolescent Medicine, Heidelberg University Hospital, 69120 Heidelberg, Germany; Franz.Schaefer@med.uni-heidelberg.de; 9 Department of Global Health and Population, Harvard T. H. Chan School of Public Health, 677 Huntington Ave, Boston, MA 02115, USA; sunghwihong@gmail.com; 10 College of Medicine, Gyeongsang National University, Jinju 52727, Korea; gwangh.jeong@gmail.com; 11 Department of Internal Medicine IV (Nephrology and Hypertension), Medical University Innsbruck, Anichstraße 35, 6020 Innsbruck, Austria; andreas.kronbichler@i-med.ac.at

**Keywords:** chronic kidney disease, end-stage renal disease, epidemiology, meta-analysis, umbrella review

## Abstract

Patients with chronic kidney disease (CKD) have altered physiologic processes, which result in different treatment outcomes compared with the general population. We aimed to systematically evaluate the efficacy of clinical interventions in reducing mortality of patients with CKD. We searched PubMed, MEDLINE, Embase, and Cochrane Database of Systematic Reviews for meta-analyses of randomized controlled trials (RCT) or observational studies (OS) studying the effect of treatment on all-cause mortality of patients with CKD. The credibility assessment was based on the random-effects summary estimate, heterogeneity, 95% prediction intervals, small study effects, excess significance, and credibility ceilings. Ninety-two articles yielded 130 unique meta-analyses. Convincing evidence from OSs supported mortality reduction with three treatments: angiotensin-converting-enzyme inhibitors or angiotensin II receptor blockers for patients not undergoing dialysis, warfarin for patients with atrial fibrillation not undergoing dialysis, and (at short-term) percutaneous coronary intervention compared to coronary artery bypass grafting for dialysis patients. Two treatment comparisons were supported by highly credible evidence from RCTs in terms of all-cause mortality. These were high-flux hemodialysis (HD) versus low-flux HD as a maintenance HD method and statin versus less statin or placebo for patients not undergoing dialysis. Most significant associations identified in OSs failed to be replicated in RCTs. Associations of high credibility from RCTs were in line with current guidelines. Given the heterogeneity of CKD, it seems hard to assume mortality reductions based on findings from OSs.

## 1. Introduction

Chronic kidney disease (CKD) is a progressive condition arising from various heterogeneous disease pathways, which results in irreversible changes of kidney function and structure [1]. The Kidney Disease: Improving Global Outcomes (KDIGO) 2012 Clinical Practice Guideline for the Evaluation and Management of Chronic Kidney Disease defines CKD as abnormalities of kidney structure or function shown by an estimated glomerular filtration rate (eGFR) of less than 60 mL/min per 1.73 m², or markers of kidney damage, present for >3 months, with implications for health [2]. CKD may lead to serious complications such as cardiovascular disease, anemia, and metabolic bone disease, which results in high cardiovascular and non-cardiovascular mortality that rises as the severity of CKD increases [3]. CKD accounted for 473.9 disease-adjusted life years lost per 100,000 population in 2016 [4], and its incidence is increasing around the world as a result of increasing life spans and a rising prevalence of hypertension, obesity, and diabetes [5].

It may be difficult to generalize findings from other populations to patients with CKD because kidney disease impacts multiple organ systems and physiologic processes in addition to altering absorption, metabolism, and excretion capabilities, which leads to a higher chance of toxin accumulation and medication interactions [1,6]. Despite CKD being one of the major risk factors for the loss of healthy life years globally, people with CKD are often excluded from trials of interventions to treat diabetes, cardiovascular diseases, and cancer [1,7]. KDIGO clinical practice guidelines provide international recommendations for the treatment of CKD and related complications [2,8,9,10,11], but few of these recommendations are supported by high-level evidence and most were largely based on expert opinions [12]. Many observational studies (OSs) have studied interventions on people with CKD to compensate for the limited data of clinical trials, but findings from OSs are methodologically limited by various biases.

To synthesize the available evidence of the efficacy of interventions in CKD patients, we performed an umbrella review of the literature. We identified meta-analyses of randomized controlled trials (RCTs) or OSs reporting the efficacy of clinical interventions on patients with CKD in terms of all-cause mortality. In addition, to assess potential biases such as reporting biases and confounding biases in statistically significant findings that might lead to false positives or inflated estimate of the association [13,14], we performed re-analyses of the identified meta-analyses including statistical tests for various biases and graded the strength of the associations’ evidence between clinical intervention and all-cause mortality of CKD patients.

## 2. Materials and Methods

This systematic review was performed according to a pre-specified protocol registered at the International Prospective Register of Systematic Reviews (PROSPERO registration: CRD42018103559). The reporting was done according to Preferred Reporting Items for Systematic Reviews and Meta-Analyses (PRISMA) guidelines (Table S1) [15].

### 2.1. Data Sources and Searches

We searched PubMed, MEDLINE, Embase, and Cochrane Database of Systematic Reviews from inception to 19 February 2019. The search was limited to articles in English. We adopted search strategies using keywords such as chronic kidney disease, end-stage renal disease, dialysis, mortality, and meta-analysis (full search strategy in PubMed and MEDLINE presented in the supplementary material). Additionally, we manually searched for potential missed articles by screening the references of relevant articles.

### 2.2. Study Selection

Two investigators (J.Y.K. and J.S.) independently screened for eligible articles by screening the title, then the abstract, and then the full text. Meta-analyses of RCTs or OSs (e.g., prospective cohort, retrospective cohort, or case-control design) that investigated the associations between certain clinical interventions and all-cause mortality of patients with CKD were thought eligible. CKD definition and staging followed those of the KDIGO 2012 Clinical Practice Guideline [2], or, alternatively, followed that of the original meta-analyses. We included studies of any clinical intervention for treating CKD, complications of CKD, or other diseases in patients with CKD. We included studies that compared an intervention of interest versus any type of control intervention such as standard therapy or placebo. We assessed all-cause mortality or survival as our outcome. We excluded studies that compared the CKD patient group and non-CKD patient group, single-arm studies, studies that assessed CKD status as a predictive variable, and studies in which patient prognostic biomarkers (such as body mass index) were exposure of interest. When two or more meta-analyses of similar design in terms of patient characteristics (e.g., CKD stages), intervention arms, and study design (RCT or OS) were identified, we identified one meta-analysis as the eligible meta-analysis for our study by adhering to the following rule. First, the meta-analysis in which individual study effect sizes were reported was thought eligible, and, second, the meta-analysis with the largest number of patients was thought eligible.

### 2.3. Data Extraction and Quality Assessment

Data extraction was performed independently by two investigators (J.Y.K. and J.S.). From eligible meta-analyses, we extracted the name of the first author, year of publication, intervention therapy, control therapy, population characteristics such as CKD stages, outcome metrics such as risk ratio (RR), odds ratio (OR), and hazard ratio (HR), summary estimate, 95% confidence interval (CI), follow-up duration, study design (RCT or OS), the number of observed deaths, and the number of total participants. From meta-analyses of OSs, we extracted the maximally adjusted summary estimate. When available, we extracted the effect estimate and its 95% CI of all component studies in the meta-analysis to perform tests for various biases and grade their level of evidence based on criteria developed over previous reviews. The eligible meta-analyses that provided these data were regarded as meta-analyses eligible for re-analysis. 

### 2.4. Data Synthesis and Analysis

For meta-analyses eligible for re-analysis, we re-performed meta-analysis under random and a fixed effects model. Statistical significance was claimed at *p*-value < 0.05. We also assessed *p*-value below thresholds of 10^−3^ and 10^−6^ [16,17]. In statistically significant associations, we checked whether the effect of the largest component study (the study with the smallest standard error) was statistically significant. Results were obtained with the identical type of metrics (RR, HR, or OR) as in the original analyses. We performed Cochran’s *Q* test and calculated the I^2^ statistic for evaluation of heterogeneity [18,19]. We estimated the 95% prediction interval, which is the range where the effect of the intervention is to be expected for 95% of similar studies in the future [20,21]. We assessed the presence of small study effects with the regression asymmetry test proposed by Egger et al. [22,23]. We performed a test for excess significance to evaluate whether the number of studies reporting nominally significant results (*p*-value < 0.05) is greater compared to their expected number [24,25]. We applied various credibility ceilings to OSs to account for their inherent methodological limitations [26,27]. All statistical tests are two-sided. Analyses used R version 3.6.2 (Vienna, Austria) and its packages (details of statistical analytic methods described in the supplementary material) [28,29,30,31].

### 2.5. Credibility Assessment

In accordance with previous umbrella reviews [32,33,34,35,36], we adopted criteria to grade the strength of the evidence of the associations between clinical interventions and all-cause mortality of CKD patients. For meta-analysis of OSs, we graded the evidence level to five categories: convincing, highly suggestive, suggestive, weak, and not significant, in terms of *p*-value under random effects, the number of deaths, statistical significance of the largest component study, heterogeneity, 95% prediction interval, estimate under credibility ceiling, and presence of biases. From meta-analyses of RCTs, we identified associations of high credibility by assessing the *p*-value under random effects, the number of deaths, the statistical significance of the largest component study, heterogeneity, a 95% prediction interval, and presence of biases. Details of criteria are provided in Table 1. 

We attempted to identify the impact of different CKD stages and potential biases arising from study designs. For overlapping meta-analyses of either OSs or RCTs that investigated the similar comparison in similar patient groups, we compared the direction and the statistical significance of the associations and assessed the difference of effect in terms of Cochran’s *Q* test for heterogeneity. In addition, for overlapping meta-analyses that investigated the similar comparison groups in a similar study design but studied patient groups of different CKD levels or dialysis statuses, we compared the direction and the statistical significance of the associations and assessed the difference of effect using Cochran’s *Q* test. A difference beyond chance between the summary estimates of the two meta-analyses was claimed at a *p*-value for Cochran’s *Q* test < 0.1. For evidence from observational studies graded as convincing or highly suggestive evidence, we performed sensitivity subset analyses by including only prospective studies, and recorded the change of the evidence level.

## 3. Results

**Figure 1:**
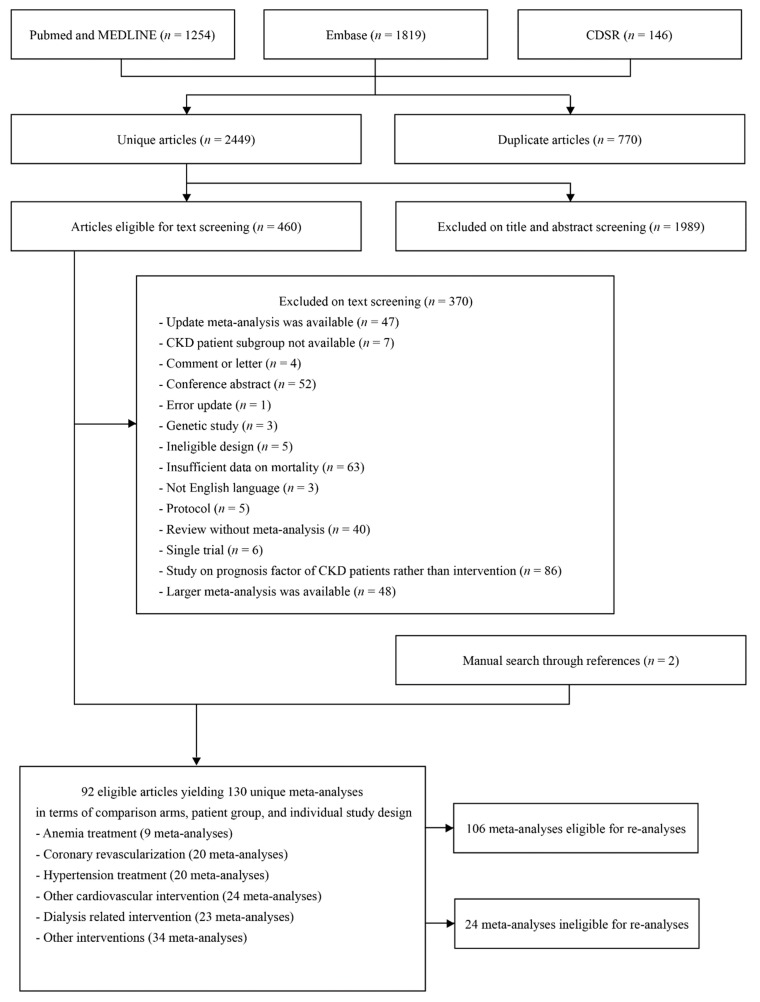
Flow chart of literature searches.

**Figure 2:**
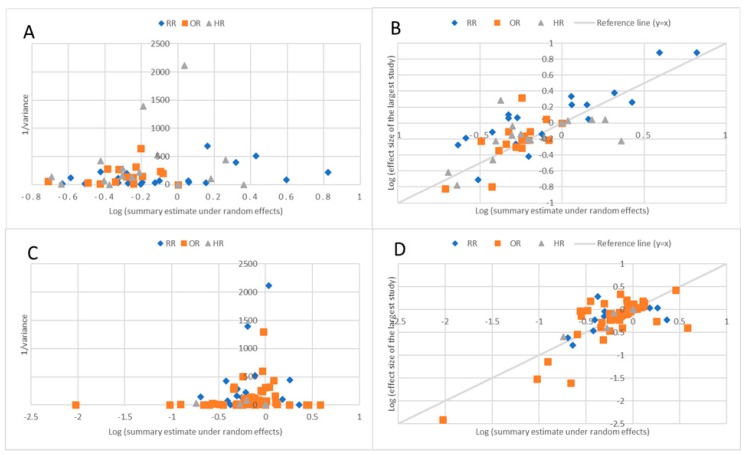
(**A**): Summary estimate of random effects, summary estimate, and inverse variance of meta-analysis of observational studies. The Y-axis labelled “Inverse variance” represents the inverse variance (1/variance) of the random effects’ summary estimate of each meta-analysis. The X-axis labelled “Log (summary estimate under random effects model)” represents the log of the summary estimate under random effects of each meta-analysis, presented. (**B**): Log (effect size of the largest study) versus log (summary effect under random effects) for each meta-analysis of observational studies. The Y-axis labelled “Log (effect size of the largest study)” represents the log of the effect estimate of the largest component study (study with the smallest standard deviation) of each meta-analysis. The X-axis labelled “Log (summary estimate under random effects model)” represents the log of the summary effect estimate under random effects of each meta-analysis. (**C**): Summary estimate of random effects summary estimate and inverse variance of meta-analysis of randomized controlled trials. (**D**): Log (effect size of the largest study) versus log (summary effect under random effects) for each meta-analysis of randomized controlled trials.

The search initially identified 3219 potentially eligible articles. After the screening process, we identified 92 articles including 130 unique meta-analyses that examined the association between a clinical intervention and all-cause mortality among patients with CKD (Figure 1). Out of 130 meta-analyses, a total of 106 meta-analyses were eligible for re-analyses (individual study effect sizes were provided). Nine meta-analyses studied management of anemia such as erythropoietin stimulating agent use, 23 meta-analyses studied dialysis methods, 20 meta-analyses studied coronary revascularization techniques, and 20 meta-analyses compared blood pressure (BP) lowering agents, while 24 meta-analyses studied other cardiovascular interventions such as implantable cardioverter defibrillator (ICD), statin, or warfarin use. A total of 106 meta-analyses were eligible for re-analysis (individual study data was available), which comprised 170,203 mortality events and a total population of 3,695,542 people. Fifty (47%) of these meta-analyses were based on OSs, while the other 56 (53%) were based on only RCTs. For meta-analyses of RCTs, the median number of study estimates was 5 (range 2–21), and the median numbers of deaths and the total participants were 230 and 1730, respectively. For meta-analyses of OSs, the median number of study estimates was 8 (range 2–26), and the median number of deaths and total population was 1,226 and 15,493. Metrics used to present the summary estimate were either RR, HR, or OR. Effect sizes of meta-analyses showed a trend toward the null value as the inverse variance of the summary estimate increased, and, among included meta-analyses, the effect sizes of the largest studies were similar to the random effects summary estimates (Figure 2).

Out of 56 eligible meta-analyses of RCTs, a total of 10 (18%) associations showed statistical significance under the random effects model, and only three (5%) had *p*-value < 10^−3^, which were warfarin for atrial fibrillation in non-hemodialysis (HD) patients, beta blockers for heart failure, and high-flux HD versus low-flux HD. Seven (13%) associations were based on 1000 or more deaths. Out of 10 statistically significant associations, seven were also supported by the statistically significant result of the largest component study, and 95% prediction intervals excluded the null in six associations. Out of statistically significant associations, only one had small study effects, and an excess significance bias was not claimed in any of the associations. 

Out of 50 eligible meta-analyses of OSs, a total of 33 (66%) associations showed statistical significance under the random effects model, 22 (44%) had a *p*-value < 10^−3^, and 15 (30%) had a *p*-value < 10^−6^. Thirty (60%) associations were based on 1000 or more deaths, of which 22 (44%) were also supported by a *p*-value < 10^−3^. Out of 33 statistically significant associations, 29 were also supported by the statistically significant result of the largest component study. Nine associations had small study effects, and two associations were claimed to have an excess of a significant bias. Heterogeneity between the studies was generally high with 34 (68%) associations having I^2^ greater than 50%. Furthermore, a 95% prediction interval excluded the null in only seven (14%) associations. While 33 (66%) associations were statistically significant under the random effects model, 28 (56%), 20 (40%), 12 (24%), and 7 (14%) retained statistical significance under 5%, 10%, 15%, and 20% credibility ceilings.

Associations between clinical interventions and all-cause mortality were graded to adhere to the pre-determined criteria (Table 1, Table 2, and Tables S2–S5). Out of associations supported by meta-analyses of OSs, three were graded as convincing evidence. These were angiotensin-converting-enzyme inhibitors (ACEI) or angiotensin II receptor blockers (ARB) as a BP-lowering agent for non-HD patients, warfarin for atrial fibrillation for non-HD patients, and short-term mortality after percutaneous coronary intervention (PCI) versus coronary artery bypass grafting (CABG) for CKD patients undergoing dialysis (Table 2). Ten associations were graded as highly suggestive evidence (Table 2). Eight associations were supported by suggestive evidence, 11 associations were supported by weak evidence, and 17 did not show statistically significant associations (Table S2). Out of associations supported by meta-analyses of RCTs, two associations were thought to have high credibility. These were high-flux HD versus low-flux HD as a maintenance dialysis method and statin versus lower-dose statin or placebo for CKD patients not on dialysis (Table 2 and Table S3). 

We identified 24 meta-analyses reporting all-cause mortality, which were unique in design but not eligible for re-analysis (Table S6). All these meta-analyses were based on RCTs. While the re-analysis was not possible, reported *p*-values were larger than the high credibility threshold of 10^−3^, so these associations did not meet criteria for high credibility. One exception was statin versus no statin or placebo for patients with CKD stage 1–2, but another eligible meta-analysis of RCTs of similar intervention, including statin versus no statin or placebo for patients not on dialysis, were already graded as having high credibility.

Meta-analyses of similar design were conducted with both RCTs and OSs in 10 comparisons (Table 3). The total number of participants in meta-analyses of OSs was generally large (range 2729 to 218,639) compared with RCTs (range 526 to 2736). Only one association was supported by statistically significant results from both RCTs and OSs (intensive versus conventional HD). One association was supported by a statistically significant result from RCTs but not from OSs, and the remaining eight associations were supported by statistically significant results from OSs but not RCTs. In eight out of ten associations, the effect heterogeneity between study designs was statistically significant. Meta-analyses of similar comparisons based on patients with different CKD stages (less severe and more severe stage) were available in 21 comparisons (Table S7). The benefit of clinical intervention on all-cause mortality was shown only in meta-analyses of less severe stage in five comparisons, and only in a meta-analysis of a more severe stage in one comparison. In two of these comparisons (statin versus less statin or placebo and ICD versus no ICD), the authors of the original meta-analyses also reported smaller relative effects on mortality as eGFR declined [37,38]. The effect difference between study designs was statistically significant in four comparisons. When sensitivity subset analyses of prospective studies were attempted on 13 meta-analyses (Table S8), the evidence was convincing for ACEI or ARB as a BP-lowering agent for non-HD patients, and the evidence was highly suggestive for warfarin for atrial fibrillation for non-HD patients and the catheter as HD access vs. fistula. 

## 4. Discussion

We identified and comprehensively analyzed 130 meta-analyses from 92 articles. While 43 associations were statistically significant, only three were supported by convincing evidence from OSs, and only two were supported by robust evidence from RCTs. Some fields of treatment studied by multiple meta-analyses were sparsely supported by statistically significant mortality benefit. Nine meta-analyses studied interventions for anemia in CKD, of which only meta-regression analyses of RCTs achieved statistical significance, which shows that a higher dose of erythropoietin stimulating agents was associated with higher mortality [39]. Out of five meta-analyses studying phosphate binders, no regimen was shown effective against placebo in terms of mortality. 

More advanced CKD is associated with a higher risk of cardiovascular mortality [40]. In patients with coronary heart disease and CKD, the ideal revascularization strategy is still unknown. Evidence from OSs suggested that CABG was convincingly associated with higher short term-mortality than PCI in CKD stage 5 patients (RR = 2.28, 95% CI = 1.99 to 2.6). There was no statistically significant difference in long-term mortality in CKD stage 5 patients. Eighteen meta-analyses of OSs compared coronary revascularization therapies for CKD patients. Comparing CABG and PCI, CABG was associated with higher short-term mortality regardless of CKD status, but was beneficial in terms of long-term mortality in patients with CKD stages 3–5 but not in dialysis patients. However, meta-analyses of three post-hoc analysis of RCTs concluded that, for patients with CKD stage 3–5, CABG compared to PCI was associated with neither short-term mortality nor long-term mortality. As guidelines suggest [41], RCTs for patients with CKD such as the ongoing International Study of Comparative Health Effectiveness with Medical and Invasive Approaches-Chronic Kidney Disease (ISCHEMIA-CKD) trial are needed to validate the optimal revascularization therapy for patients with CKD. 

Convincing evidence from OSs also suggested that ACEI or ARB were associated with lower mortality in CKD patients not on dialysis. Meta-analyses of RCTs also suggested that, compared to placebo, ACEI was associated with lower mortality, while ARB was not. However, whether these findings lead to superiority of ACEI over ARB is uncertain because trials of ACEIs tended to be outdated than trials of ARBs, and head-to-head trials failed to show a statistically significant difference on mortality [42]. In CKD patients with diabetes, use of ACEI nor ARB was not associated with lower mortality, even though it did lower the risk of progression to end-stage renal disease (ESRD) [43]. KDIGO guidelines indicate that the choice of BP lowering agents for CKD patients is less important than the achieved BP reduction. The guidelines also indicate that there is no strong evidence to support the preferential use of any agents except for use of ACEI or ARB for CKD patients with albuminuria [9]. Accordingly, meta-analyses of RCTs found that more intensive BP control was associated with lower mortality rates in CKD patients with or without dialysis [44,45], even though *p*-values were close to the significance threshold. 

Warfarin was associated with lower mortality in non-HD patients with atrial fibrillation, which is supported by convincing evidence from OSs. Meta-analysis of RCTs was not available. A post-hoc analysis of RCT reported reduced ischemic stroke/systemic embolism events in patients with CKD stage 3 using adjusted-dose warfarin compared to aspirin/low-dose warfarin [46]. Based on findings from RCTs, guidelines recommend the use of vitamin K antagonist or new oral anticoagulants for non-HD patients with atrial fibrillation [47,48]. However, for dialysis patients with atrial fibrillation, no RCT of anticoagulant therapies was available [47,48]. This was reflected in discordant recommendations, whereby one guideline did not recommend anticoagulant use for dialysis patients [47], and the other recommended warfarin but not new oral anticoagulants based on observational data [48]. However, the mortality benefit of warfarin in dialysis patients did not reach statistical significance in a meta-analysis of OSs [49]. In RCTs of anticoagulants, people with CKD and ESRD are frequently excluded [50,51]. Further well-conducted RCTs are needed to reach a clear conclusion. 

Highly credible evidence from RCTs showed that high-flux HD was associated with lower mortality compared with low-flux HD. Guidelines recommended both high-flux HD and low-flux HD as maintenance dialysis methods, while acknowledging a moderate benefit of high-flux HD on several secondary outcomes with no increase in harm but also high cost and low availability [52]. Highly credible evidence from RCTs also showed that statin use was associated with lower all-cause mortality in CKD patients not on dialysis, but the association was not significant in patients undergoing dialysis. An individual patient data meta-analysis also showed that there were trends for relatively smaller cardiovascular and all-cause mortality benefits of statins as eGFR declined [53]. The benefit of statin use in CKD patients not on dialysis was also acknowledged by current guidelines [11,54,55]. The KDIGO guidelines recommend statin treatment for adults aged >50 years with CKD not on dialysis but did not recommend statin therapy initiation in dialysis-dependent CKD patients [11].

When meta-analyses of RCTs and OSs with a similar design (in terms of comparison arms, CKD stage, and underlying comorbidities) were compared, only one out of nine statistically significant associations from OSs were replicated in RCTs, while the heterogeneity of effects between study designs was statistically significant in eight out of ten associations. Compared with eligible meta-analyses of RCTs, a higher percentage of meta-analyses of OSs reported significant associations but had large heterogeneity or signs of small study effects. These differences could have been caused by patient group heterogeneity among the individual studies, and a relatively low number of patients or a selection bias in RCTs. Residual confounding among observational studies is also likely. When estimating a treatment effect using observational data, it is very difficult to fully adjust for all potential biases including confounding by indication [56]. The high rate of complications in CKD patients may also have acted as underlying causes of confounding biases. 

Numerous guidelines highlighted the importance of specific treatment recommendations for patients with CKD due to their high rate of morbidities and high mortality [41,47,48,57]. This is especially true for interventions targeting cardiovascular disease [40] since rates of cardiovascular-related adverse events are strongly and independently related to the CKD stage. Yet, most of the recommendations for CKD patients were based on low evidence from observational studies or expert opinions due to a lack of trial data, with the latter being a particular issue for patients with ESRD on dialysis who are frequently excluded [51]. We observed a substantial discrepancy in the observed effect size between the study designs (RCT or OS) that may make it hard to base treatment decisions for CKD patients on findings from OSs alone, which are more susceptible to numerous biases due to their innate methodological limitations. To overcome the exclusion of patients with non-severe and severe CKD, trials including only CKD patients should be conducted to assess the true effect of the treatment on CKD patients such as the ongoing ISCHEMIA-CKD trial or the recently published Canagliflozin and Renal Outcomes in the Type 2 Diabetes and Nephropathy (CREDENCE) trial [58]. Alternatively, future trials, especially trials assessing cardiovascular interventions, should include adequate numbers of patients with CKD of various stages to assess if any treatment effect is modified by the CKD status. Additionally, the reporting of relationships between the severity of CKD and benefit of treatment, such as those reported in several meta-analyses of cardiovascular interventions [37,38], might be helpful to assume treatment effect in CKD patients of a severe stage when data from trials are not yet available.

One notable limitation of our study is that we only searched for meta-analyses in our study, and large individual trials might have been missed. We could not identify new, large trials comparing statin use versus non-statin use, but concurrent meta-analyses all showed a consensus in that statin therapy lowered mortality in non-dialysis patients [59,60]. We identified a few trials comparing high-flux HD and low-flux HD [61,62]. While these trials were not powered enough to assess a mortality benefit in either modality, the results of these trials were consistent with the results of our re-analysis in that high-flux dialysis were associated with lower FGF-23 and β2m level. This signifies better middle molecular clearance in patients with ESRD. Additionally, while we were not able to identify meta-analyses of RCTs eligible for re-analyses studying two of the associations graded as convincing evidence by observational studies (ACEI or ARB vs. no ACEI or ARB for patients not undergoing dialysis and warfarin for atrial fibrillation vs. no warfarin for patients not undergoing dialysis), we found that results from RCTs showed consistent results within these two associations. A meta-analysis showed that ACEI or ARB showed reduced renal failure in patients with non-diabetic CKD [63], and a network meta-analysis showed that ACEIs showed reduced mortality against active controls in CKD patients with or without diabetes [64]. A retrospective analysis of CKD stage 3 subgroup of two trials showed that an adjusted-dose warfarin group showed statistically significant 24% reduction in ischemic stroke or systemic embolism compared to low-dose warfarin or heparin group, even though a mortality benefit was not significant [46]. These findings support the robustness of the two associations of convincing evidence.

Some other limitations also exist. We did not assess the individual quality of the included meta-analyses, as this was the responsibility of the original authors. Although we adopted evidence grading criteria applied and developed for numerous previous studies, the criteria are mainly based on arbitrary thresholds. Although we applied the most rigorous criteria to control the potential biases in literature, we cannot be fully sure that results thought as credible in our review are indeed genuine. Fourth, we assessed only all-cause mortality of the reported associations. While doing so allowed us to objectively assess the comparative efficacy of various treatments and avoid reporting a large number of nominally significant associations, other treatments shown to improve secondary outcomes such as progression of CKD and cardiovascular events could have been missed. Non-significant mortality associations may not necessarily imply that treatment is not beneficial, and further well-designed studies are warranted. This relates particularly to associations supported by studies with a short follow-up period reporting a benefit in secondary outcomes.

## 5. Conclusions

Despite the limitations, we have identified the available evidence regarding the efficacy of a vast range of treatments for patients with CKD. While the associations from RCTs graded as having high credibility were in line with recommendations from current guidelines, treatment efficacy supported by OSs was not consistent with results from RCTs. It may be hard to assume the benefit of treatment on mortality based on findings from OSs. Future trials focusing on CKD patients are warranted to provide optimized treatment strategies to this cohort with a high unmet need.

## Figures and Tables

**Table 1:** Summary of evidence grading for associations between clinical intervention and all-cause mortality of patients with chronic kidney disease (for associations having *p*-value < 0.05).

All-Cause Mortality Evidence Category	Clinical Interventions, CKD Patient Group ^a^	Coronary Revascularization-Related Interventions, Studied Outcome, CKD Patient Group ^a^
**Observational studies**		
Convincing evidence *p*-value < 10^−6^ under random effects, >1000 observed deaths, *p*-value < 0.05 of the largest study in meta-analysis, no signs of bias, ^b^ statistical significance was retained in 10% credibility ceiling, 95% prediction interval excludes the null	ACEI or ARB vs. control, CKD stage ND; warfarin for atrial fibrillation vs. no warfarin, CKD stage ND	PCI vs. CABG, short-term acm, CKD stage 5D
Highly suggestive evidence *p*-value < 10^−6^ under random effects, >1000 observed deaths, *p*-value < 0.05 of the largest study in meta-analysis	Early vs. late angiography for non ST elevation acute coronary syndrome, any CKD stage, ICD for heart failure, CKD stage 5D, vitamin D, CKD stage 5D, late vs. early dialysis initiation, fistula vs. catheter as HD access route, graft vs. catheter as HD access route, intensive vs. conventional HD, CKD stage 5, early referral to professional nephrology service, any CKD stage, influenza vaccine, CKD stage 5	CABG vs. PCI, long-term acm, CKD stage < 5; PCI vs. CABG, short-term acm, CKD stage 3–5
Suggestive evidence *p*-value < 10^−3^ under random effects, >1000 observed deaths	ICD for heart failure, CKD stage 3–5, parathyroidectomy for secondary hyperparathyroidism, CKD stage 5D, fistula vs. graft as HD access, intensive HD vs. PD, healthy dietary pattern, CKD stage 3–5	PCI vs. medical therapy, long-term acm, CKD stage 3–5, DES vs. BMS, long-term acm, any CKD stage, DES vs. BMS, long-term acm, CKD stage 5D
Weak evidence *p*-value < 0.05 under random effects	Combined vs. single RAAS blockade, CKD stage 5, metformin for type 2 diabetes, CKD stage 3–5, statin vs. less statin or placebo, CKD stage 5D with diabetes, vitamin D, CKD stage ND, dialysis vs. conservative therapy, CKD stage 5, HD vs. PD, multidisciplinary care, any CKD stage	Off pump CABG vs. on pump CABG, short-term acm, CKD stage 3–5; PCI vs. medical therapy, short-term acm, CKD stage 5D, 2nd generation DES vs. 1st generation DES, long-term acm, CKD stage > 3, DES vs. BMS, short-term acm, CKD stage 5D; CABG vs. PCI, long-term acm, CKD stage 3–5
**Randomized controlled trials**		
*p*-value < 10^−3^ under random effects, >1000 observed deaths, *p*-value < 0.05 of the largest study in meta-analysis, no signs of bias, ^b^ 95% prediction interval excludes the null	Statin vs. less statin or placebo, CKD ND, high-flux HD vs. low-flux HD, CKD stage 5	Not available
*p*-value < 0.05 under random effects	Beta-blockers for heart failure, CKD stage 3–5, more intensive vs. less intensive blood pressure target, CKD stage 3–5ND, more intensive vs. less intensive blood pressure target, CKD stage 5D, mineralocorticoid receptor antagonist, any CKD stage, mineralocorticoid receptor antagonist, CKD stage 5, ICD for heart failure, CKD stage 1–2, lanthanum carbonate vs. other phosphate-binding agents, CKD stage 5HD, non-calcium-based phosphate binders vs. calcium-based phosphate binders, CKD stage 3–5	Not available

^a^. In comparison a vs. b, a arm is beneficial than b arm in terms of all-cause mortality. b. Not large heterogeneity, no signs of small study effects, and no signs of excess significance bias. Heterogeneity was assessed in terms of Cochran’s *Q* test and large heterogeneity was defined as I2 statistic > 50%. Small study effects were assessed by Egger’s asymmetry test and were claimed at Egger *p*-value < 0.1. Excess significance bias was assessed with the largest individual study (smallest standard error) as a plausible effect size of meta-analysis, and was claimed at *p*-value < 0.1 with a number of observed studies larger than the number of expected studies. All statistical tests are two-sided. Abbreviations: ACEI, angiotensin-converting enzyme inhibitor. acm, all-cause mortality. ARB, angiotensin receptor blocker. BMS, bare metal stent. CABG, coronary artery bypass. CKD, chronic kidney disease. DES, drug-eluting stent. HD, hemodialysis. ICD, implantable cardioverter defibrillator. PCI, percutaneous intervention. PD, peritoneal dialysis. RAAS, renin–angiotensin–aldosterone system. vs., versus.

**Table 2:** Details of meta-analyses associating clinical intervention and all-cause mortality of patients with chronic kidney disease (presented were associations graded as convincing or highly suggestive for meta-analysis of observational studies, and associations having *p*-value < 0.05 for meta-analysis of randomized controlled trials).

Author, Year	Comparison (Experimental Arm vs. Control Arm)	CKD Stage	Follow−Up Duration (Months) ^a^ or Time of Outcome Measurement	Number of Studies	Deaths/Population	Effect Metrics	Summary Effect Estimate (95% CI) under Random Effects ^b^	Summary Estimate *p*−Value	I^2^ (%)	95% Prediction Interval	Evaluation of Bias ^c^
**Observational studies, convincing evidences**											
Volodarskiy et al., 2016	CABG vs. PCI	5D	Short−term mortality (in−hospital or at 30 days)	11	3347/52,192	RR	2.28 (1.99 to 2.6)	4.5 × 10^−34^	40	1.69 to 3.06	None
Qin et al., 2016	ACEI or ARB vs. no ACEI or ARB	ND	12–96	10	>1000/81,959	HR	0.83 (0.78 to 0.87)	9.8 × 10^−13^	44	0.73 to 0.94	None
Dahal et al., 2016	Warfarin for atrial fibrillation vs. no warfarin	ND	29–180	4	>1000/30,333	HR	0.66 (0.6 to 0.72)	3.4 × 10^−18^	39	0.47 to 0.91	None
**Observational studies, highly suggestive evidences**											
Kannan et al., 2016	CABG vs. PCI	<5	34	7	4327/15,493	OR	0.82 (0.76 to 0.88)	2.8 × 10^−7^	0	0.74 to 0.9	Loss of significance under 10% credibility ceiling
Volodarskiy et al., 2016	CABG vs. PCI	3–5	Short−term mortality (in−hospital or at 30 days)	14	3470/55,068	RR	1.81 (1.47 to 2.24)	4.0 × 10^−8^	75	0.98 to 3.36	Large heterogeneity, loss of significance under 10% credibility ceiling
Shaw et al., 2016	Early invasive coronary angiography and/or revascularization for non−ST elevation acute coronary syndrome vs. initial conservative approach	Any	Mortality assessed in−hospital or at 6–12 months	9	>1000/147,908	HR	0.5 (0.42 to 0.59)	1.4 × 10^−16^	79	0.3 to 0.85	Large heterogeneity
Shurrab et al., 2018	ICD for primary prevention of sudden cardiac death vs. no ICD	5D	11–56	4	4366/6485	OR	0.49 (0.38 to 0.63)	3.4 × 10^−8^	17	0.23 to 1.06	Loss of significance under 10% credibility ceiling
Lu et al., 2017	Vitamin D or analogues vs. non−vitamin D treatment	5D	4–121	16	>1000/218,639	RR	0.65 (0.57 to 0.75)	1.9 × 10^−10^	94	0.41 to 1.05	Large heterogeneity, small study effects
Zhao et al., 2018	Earlier HD vs. later HD	5	12−180	10	>1000/NR	HR	1.3 (1.18 to 1.43)	3.6 × 10^−8^	98	0.97 to 1.74	Large heterogeneity, small study effects
Ravani et al., 2013	Catheter as HD access vs. fistula	5HD	18	19	>1000/411,068	RR	1.53 (1.41 to 1.67)	4 × 10^−22^	83	1.13 to 2.08	Large heterogeneity, small study effects
Ravani et al., 2013	Catheter as HD access vs. graft	5HD	18	15	>1000/394,922	RR	1.38 (1.25 to 1.52)	2.2 × 10^−10^	85	0.99 to 1.91	Large heterogeneity
Jin et al., 2013	Prolonged nocturnal or daytime HD vs. conventional HD	5	12–132	13	>1000/85,722	OR	0.72 (0.64 to 0.81)	6.9 × 10^−8^	68	0.5 to 1.03	Large heterogeneity, small study effects
Smart et al., 2014	Early referral to specialist nephrology services vs. late referral to specialist nephrology services	Any	12	16	4030/23,238	RR	0.56 (0.47 to 0.66)	8.1 × 10^−11^	82	0.3 to 1.02	Large heterogeneity, small study effects
Remschmidt et al., 2014	Influenza vaccine vs. control	5	NR	4	>1000/174,663	OR	0.68 (0.6 to 0.76)	9.1 × 10^−11^	83	0.41 to 1.13	Large heterogeneity
**Randomized controlled trials ( >1000 observed deaths; *p*−value < 0.05 of the largest study in meta−analysis, no signs of bias 95% prediction interval excludes the null)**											
Zhang et al., 2014	Statin vs. less statin or placebo	ND	23–64	7	2351/33,589	RR	0.78 (0.72 to 0.86)	4.2 × 10^−8^	5	0.68 to 0.9	None
Tan et al., 2018	High−flux HD vs. low−flux HD	5	24–72	9	>1000/8385	RR	0.71 (0.63 to 0.8)	8.5 × 10^−9^	0	0.62 to 0.82	None
**Randomized controlled trials, *p*−value < 0.05**											
Badve et al., 2011	Beta−blockers for heart failure vs. placebo	3–5	12–24	5	980/5702	RR	0.72 (0.64 to 0.8)	2.6 × 10^−9^	0	0.6 to 0.86	None
Malhotra et al., 2017	More intensive vs. less intensive blood pressure target	3–5ND	43	17	1293/15,914	OR	0.86 (0.76 to 0.96)	0.01	0	0.76 to 0.97	Small study effects
Heerspink et al., 2009	More intensive vs. less intensive blood pressure target	5D	12–36	7	481/1571	RR	0.8 (0.66 to 0.96)	0.015	31	0.53 to 1.2	None
Lu et al., 2016	Spironolactone or eplerenone vs. no mineralocorticoid receptors	Any	3–56	5	NR/1724	RR	0.58 (0.36 to 0.91)	0.018	49	0.15 to 2.14	None
Quach et al., 2016	Spironolactone or eplerenone vs. placebo or none	5	3–36	6	59/721	RR	0.4 (0.23 to 0.7)	0.0012	0	0.19 to 0.88	None
Pun et al., 2014	ICD for primary prevention of sudden cardiac death vs. no ICD	1	20–40	3	NR/NR	HR	0.48 (0.34 to 0.67)	1.7 × 10^−5^	0	0.05 to 4.24	None
Wang et al., 2018	Lanthanum carbonate vs. calcium−based phosphate binders or sevelamer	5HD	5–24	6	171/1730	OR	0.45 (0.32 to 0.63)	2.9 × 10^−6^	0	0.28 to 0.72	None
Sekercioglu et al., 2016	Non−calcium−based phosphate binders vs. calcium−based phosphate binders	3–5	>1	15	NR/NR	RR	0.57 (0.39 to 0.83)	0.003	72	0.2 to 1.61	Large heterogeneity

^a^. Represented as median or range of follow-up duration of individual studies. b. Summary estimate smaller than 1 favors experimental arm (lower mortality in experimental arm), effect estimate larger than 1 favors control arm (lower mortality in the control arm). c. Any of the following: large heterogeneity, signs of small study effects, signs of excess significance bias, and for observational studies, loss of statistical significance in a 10% credibility ceiling. All statistical tests are two-sided. Abbreviations: ACEI, angiotensin-converting enzyme inhibitor. ARB, angiotensin receptor blocker. CABG, coronary artery bypass. CI, confidence interval. CKD, chronic kidney disease. HD, hemodialysis. HR, hazard ratio. ICD, implantable cardioverter defibrillator. NR, not reported. OR, odds ratio. PCI, percutaneous intervention. RR, risk ratio. vs., versus.

**Table 3:** Comparisons of effect of treatment on all-cause mortality between evidences from observational studies and randomized controlled trials.

Comparison (Experimental Arm vs. Control Arm)	CKD Stage	Observational Studies			Randomized Controlled Trials			*p*-value for Heterogeneity ^b^	Statistical Significance
		Effect Metric	Random Effects Summary Estimate (95% CI) ^a^	Deaths/ Population	Effect Metric	Random Effects Summary Estimate (95% CI) ^a^	Deaths/ Population		
CABG vs. PCI, long-term acm	ND	OR	0.82 (0.76 to 0.88)	4327/15,493	HR	0.99 (0.67 to 1.46) ^c^	NR/526	0.34	Only OSs
CABG vs. PCI, short-term acm	ND	RR	1.81 (1.47 to 2.24)	3470/55,068	HR	0.92 (0.54 to 1.58) ^c^	NR/526	0.021	Only OSs
DES vs. BMS, long-term acm	Any	OR	0.79 (0.71 to 0.89)	>1000/117,247	RR	0.99 (0.78 to 1.27)	230/1567	0.1	Only OSs
Mineralocorticoid receptor antagonist vs. control	Any	RR	0.9 (0.71 to 1.15)	NR/2863	RR	0.58 (0.36 to 0.91)	NR/1724	0.089	Only RCTs
Early vs. late angiography for non ST elevation acute coronary syndrome	Any	HR	0.5 (0.42 to 0.59)	>1000/147,908	HR	0.76 (0.49 to 1.17)	NR/1453	0.076	Only OSs
Statin vs. less statin or placebo	5D with diabetes	HR	0.67 (0.49 to 0.93)	NR/11,095	HR	0.9 (0.8 to 1.02)	NR/1986	0.096	Only OSs
Vitamin D vs. control	5D	RR	0.65 (0.57 to 0.75)	>1000/218,639	RR	1.13 (0.63 to 2.03)	NR/700	0.075	Only OSs
Vitamin D vs. control	ND	RR	0.53 (0.32 to 0.87)	NR/2729	RR	1.55 (0.52 to 4.62)	NR/832	0.082	Only OSs
Intensive HD vs. conventional HD	5	OR	0.72 (0.64 to 0.81)	>1000/85,722	HR	0.86 (0.75 to 0.99) ^c^	769/2736	0.061	Both significant in same direction
Multidisciplinary care vs. control	Any	OR	0.61 (0.43 to 0.86)	762/7390	OR	0.82 (0.53 to 1.27)	240/1912	0.29	Only OSs

^a^. Summary estimate smaller than 1 favors experimental arm (lower mortality in experimental arm). Effect estimate larger than 1 favors control arm (lower mortality in control arm). b. Significance threshold of Cochran’s *Q* test for heterogeneity is *p* value < 0.1. Significant associations were shown in bold. c. Summary estimate from individual patient data meta-analyses. All statistical tests are two-sided. Abbreviations: acm, all-cause mortality. BMS, bare metal stent. CABG, coronary artery bypass. CI, confidence interval. CKD, chronic kidney disease. DES, drug-eluting stent. HD, hemodialysis. HR, hazard ratio. NR, not reported. OR, odds ratio. OS, observational study. PCI, percutaneous intervention. RCT, randomized controlled trial. RR, risk ratio. vs., versus.

## Supplementary Materials

The following are available online at https://www.mdpi.com/2077-0383/9/2/394/s1. Supplementary Method S1: PubMed and MEDLINE search strategy. Supplementary Method S2: Details of data analytic methods. Table S1: PRISMA Checklist. Table S2: Details of meta-analyses of observational studies associating clinical intervention and all-cause mortality of patients with chronic kidney disease graded as suggestive evidence, weak evidence, or not significant. Table S3: Details of meta-analyses of randomized controlled trials associating clinical intervention and all-cause mortality of patients with chronic kidney disease, having a *p*-value > 0.05. Table S4: Details of credibility assessment in meta-analyses of observational studies associating clinical intervention and all-cause mortality of patients with chronic kidney disease. Table S5: Details of credibility assessment in meta-analyses of randomized controlled trials associating clinical intervention and all-cause mortality of patients with chronic kidney disease. Table S6: Details of eligible meta-analysis unique in design but ineligible for re-analysis. Supplementary references. Table S7: Comparisons of effect of treatment on all-cause mortality between evidences from different chronic kidney disease stages. Table S8: Sensitivity subset analysis of prospective studies only of evidence from observational studies graded as convincing or highly suggestive evidence.
