# Supplementary material for: Clinical Interventions and All-Cause Mortality of Patients with Chronic Kidney Disease: An Umbrella Systematic Review of Meta-Analyses
Source: J Clin Med. 2020 Feb 1;9(2):394. doi: 10.3390/jcm9020394 (PMC7074128; doi:10.3390/jcm9020394)
Supplement: Supplementary file 1 [file jcm-09-00394-s001.pdf]

Supplementary material

Clinical interventions and all-cause mortality of patients with chronic kidney disease: an umbrella systematic review of meta-analyses

Contents

PubMed and MEDLINE search strategy

Details of data analytic methods

Table S1. PRISMA Checklist

Table S2. Details of meta-analyses of observational studies associating clinical intervention and all-cause mortality of patients with chronic kidney disease graded as suggestive evidence, weak evidence, or not significant

Table S3. Details of meta-analyses of randomized controlled trials associating clinical intervention and all-cause mortality of patients with chronic kidney disease, having *p*-value>0.05

Table S4. Details of credibility assessment in meta-analyses of observational studies associating clinical intervention and all-cause mortality of patients with chronic kidney disease

Table S5. Details of credibility assessment in meta-analyses of randomized controlled trials associating clinical intervention and all-cause mortality of patients with chronic kidney disease

Table S6. Details of eligible meta-analysis unique in design but ineligible for re-analysis

Table S7. Comparisons of effect of treatment on all-cause mortality between evidences from different chronic kidney disease stages

Table S8. Sensitivity subset analysis of prospective studies only of evidence from observational studies graded as convincing or highly suggestive evidence

Reference of eligible articles

PubMed and MEDLINE search strategy

Last search performed in: 02/19/2019

#1 "chronic kidney disease"[All Fields]
#2 "Chronic Kidney Disease-Mineral and Bone Disorder"[Mesh]
#3 "chronic kidney failure"[All Fields]
#4 "chronic renal disease"[All Fields]
#5 "chronic renal failure"[All Fields]
#6 "chronic renal insufficiency"[All Fields]
#7 "chronic"[All Fields] AND "kidney"[All Fields] AND "disease"[All Fields]
#8 "chronic"[All Fields] AND "renal"[All Fields] AND "disease"[All Fields]
#9 "chronic"[All Fields] AND "renal"[All Fields] AND "failure"[All Fields]
#10 "dialysis"[All Fields]
#11 "dialysis"[MeSH]
#12 "end stage renal disease"[All Fields]
#13 "end"[All Fields] AND "stage"[All Fields] AND "renal"[All Fields] AND "disease"[All Fields]
#14 "esrd"[All Fields]
#15 "haemodialysis"[All Fields]
#16 "hemodialysis"[All Fields]
#17 "kidney failure, chronic"[MeSH]
#18 "kidney"[All Fields] AND "failure"[All Fields] AND "chronic"[All Fields]
#19 "renal dialysis"[Mesh]
#20 "Renal Insufficiency, Chronic"[Mesh]
#21 "renal"[All Fields] AND "insufficiency"[All Fields] AND "chronic"[All Fields]
#22 CKD [All Fields]
#23 CRF [All Fields]
#24 #1 OR #2 OR #3 OR #4 OR #5 OR #6 OR #7 OR #8 OR #9 OR #10 OR #11 OR #12 OR #13 OR #14 OR #15 OR #16 OR #17 OR #18 OR #19 OR #20 OR #21 OR #22 OR #23
#25 "mortality"[All Fields]
#26 "mortality"[MeSH]
#27 “mortality"[Subheading]
#28 "death"[All Fields]
#29 "death"[MeSH]
#30 "survival"[All Fields]
#31 "survival"[MeSH]
#32 "survival rate"[Mesh]
#33 #25 OR #26 OR #27 OR #28 OR #29 OR #30 OR #31 OR #32
#34 "meta"[All Fields]
#35 "Meta-Analysis"[Publication Type]
#36 #34 OR #35
#38 #24 AND #33 AND #36

Details of data analytic methods

Assessment of heterogeneity

We performed Cochran's Q test and calculated the I2 statistic for evaluation of heterogeneity 1,2. I2 ranges from 0% to 100% and describes the percentage of variability in a study estimate that is due to between-study heterogeneity. I2 > 50% was regarded as large heterogeneity. Significant heterogeneity indicates presence of genuine heterogeneity or bias.

Estimation of the prediction interval

We estimated the 95% prediction interval, which is the range where a true effect of the intervention is to be expected for 95% of similar studies in the future 3. While the summary effects of random-effects meta-analysis represent the average effect of included studies, prediction interval estimates the treatment effect of individual studies in future settings 4. For example, a 95% prediction interval of risk ratio = (2 to 4) implies that 95% of future studies are expected to show a risk ratio between 2 and 4. Prediction intervals centers around random effects summary estimate, similar to confidence intervals. 95% prediction intervals corresponds to 95% confidence intervals when there is no in-between study heterogeneity and gets wider as in-between study heterogeneity increases. Prediction intervals including the null value suggests there may be settings where the intervention effect is null or even in the opposite direction and requires further study for identification of the causes of heterogeneity. 95% prediction interval excluding the null suggests that the treatment effect is beneficial in at least 95% of the future studies and concludes that results of treatment effects are consistent, even when some between-study heterogeneity is present.

Assessment of small study effects

We assessed small study effects, i.e. large studies having more conservative results than smaller studies, with the regression asymmetry test proposed by Egger, et al 5. Small-study effects were claimed at Egger p value < 0.1 with the effect of the largest study (the study with the smallest standard error) showing more conservative result than the summary effect of the meta-analysis under random model. Presence of small study indicates publication bias, selective reporting, or genuine heterogeneity 6.

Assessment of excess significance bias

We performed a test for excess significance to evaluate whether the number of studies reporting nominally significant results (p value < 0.05) is greater compared to the expected number of statistically significant studies 7. We assumed that the effect size of the largest study in a meta-analysis was plausible effect size of the individual studies 8. The expected probability that an individual study is statistically significant was assumed to be the power of the largest study at type I error rate = 0.05. Statistic A was calculated by the following χ2 statistic: A = {(O – E)2/E + (O – E)2/(N – E)}~ χ2, where O is the number of observed statistically significant studies, E is the expected number of statistically significant studies, and N is the total number of individual studies. Excess significance was claimed at p value < 0.1 with the number of observed significant studies larger than the number of expected significant studies. Presence of excess significance indicates publication bias, selective analysis, or outcome reporting bias.

Application of credibility ceilings

We applied credibility ceilings to observational studies to account for their inherent methodological limitations that might result in spurious significant results of meta-analyses 9,10. We assumed that every observational study could not give more than a maximum certainty of 100 - c% (c, credibility ceiling) that the effect estimate is in the direction suggested by the point estimate and not in the other. For every observational study showing the certainty higher than the allowed threshold under the given credibility ceiling, we inflated its effect variance which resulted in lower certainty which fit the threshold. We obtained random effects summary estimates and heterogeneity of meta-analyses of observational studies under 5%, 10%, 15%, and 20% credibility ceilings, and assessed whether statistical significance under random effects ($p < 0.05$) were retained.

References

1. Cochran WG. The combination of estimates from different experiments. Biometrics. 1954;10:101-29.
2. Ioannidis JP, Patsopoulos NA, Evangelou E. Uncertainty in heterogeneity estimates in meta-analyses. BMJ. 2007;335(7626):914-6.
3. Higgins JP, Thompson SG, Spiegelhalter DJ. A re-evaluation of random-effects meta-analysis. J R Stat Soc Ser A Stat Soc. 2009;172(1):137-59.
4. Riley RD, Higgins JP, Deeks JJ. Interpretation of random effects meta-analyses. BMJ. 2011;342:d549.
5. Egger M, Davey Smith G, Schneider M, Minder C. Bias in meta-analysis detected by a simple, graphical test. BMJ. 1997;315(7109):629-34.
6. Sterne JA, Sutton AJ, Ioannidis JP, et al. Recommendations for examining and interpreting funnel plot asymmetry in meta-analyses of randomised controlled trials. BMJ. 2011;343:d4002.
7. Ioannidis JP, Trikalinos TA. An exploratory test for an excess of significant findings. Clin Trials. 2007;4(3):245-53.
8. Ioannidis JPA. Clarifications on the application and interpretation of the test for excess significance and its extensions. J Math Psychol. 2013;57(5):184-7.
9. Salanti G, Ioannidis JP. Synthesis of observational studies should consider credibility ceilings. J Clin Epidemiol. 2009;62(2):115-22.
10. Papatheodorou SI, Tsilidis KK, Evangelou E, Ioannidis JP. Application of credibility ceilings probes the robustness of meta-analyses of biomarkers and cancer risk. J Clin Epidemiol. 2015;68(2):163-74.

Table S1. PRISMA Checklist

Section/topic	#	Checklist item	Reported on page #
**TITLE**			
Title	1	Identify the report as a systematic review, meta-analysis, or both.	1
**ABSTRACT**			
Structured summary	2	Provide a structured summary including, as applicable: background; objectives; data sources; study eligibility criteria, participants, and interventions; study appraisal and synthesis methods; results; limitations; conclusions and implications of key findings; systematic review registration number.	1
**INTRODUCTION**			
Rationale	3	Describe the rationale for the review in the context of what is already known.	2
Objectives	4	Provide an explicit statement of questions being addressed with reference to participants, interventions, comparisons, outcomes, and study design (PICOS).	2
**METHODS**			
Protocol and registration	5	Indicate if a review protocol exists, if and where it can be accessed (e.g., Web address), and, if available, provide registration information including registration number.	2
Eligibility criteria	6	Specify study characteristics (e.g., PICOS, length of follow-up) and report characteristics (e.g., years considered, language, publication status) used as criteria for eligibility, giving rationale.	2-3
Information sources	7	Describe all information sources (e.g., databases with dates of coverage, contact with study authors to identify additional studies) in the search and date last searched.	2-3
Search	8	Present full electronic search strategy for at least one database, including any limits used, such that it could be repeated.	2-3, supplementary material
Study selection	9	State the process for selecting studies (i.e., screening, eligibility, included in systematic review, and, if applicable, included in the meta-analysis).	2-3
Data collection process	10	Describe method of data extraction from reports (e.g., piloted forms, independently, in duplicate) and any processes for obtaining and confirming data from investigators.	3
Data items	11	List and define all variables for which data were sought (e.g., PICOS, funding sources) and any assumptions and simplifications made.	3
Risk of bias in individual studies	12	Describe methods used for assessing risk of bias of individual studies (including specification of whether this was done at the study or outcome level), and how this information is to be used in any data synthesis.	3-4
Summary measures	13	State the principal summary measures (e.g., risk ratio, difference in means).	3-4
Synthesis of results	14	Describe the methods of handling data and combining results of studies, if done, including measures of consistency (e.g., I^2) for each meta-analysis.	3-4, supplementary material

Section/topic	#	Checklist item	Reported on page #
Risk of bias across studies	15	Specify any assessment of risk of bias that may affect the cumulative evidence (e.g., publication bias, selective reporting within studies).	3-4
Additional analyses	16	Describe methods of additional analyses (e.g., sensitivity or subgroup analyses, meta-regression), if done, indicating which were pre-specified.	3-4
**RESULTS**			
Study selection	17	Give numbers of studies screened, assessed for eligibility, and included in the review, with reasons for exclusions at each stage, ideally with a flow diagram.	4,7,8, Figure 1, Supplementary material
Study characteristics	18	For each study, present characteristics for which data were extracted (e.g., study size, PICOS, follow-up period) and provide the citations.	4,7-12
Risk of bias within studies	19	Present data on risk of bias of each study and, if available, any outcome level assessment (see item 12).	7-8, Table 2, Table S2-S6
Results of individual studies	20	For all outcomes considered (benefits or harms), present, for each study: (a) simple summary data for each intervention group (b) effect estimates and confidence intervals, ideally with a forest plot.	7-8, Table 2, Table S2-S6
Synthesis of results	21	Present results of each meta-analysis done, including confidence intervals and measures of consistency.	7-8, Table 2, Table S2-S5
Risk of bias across studies	22	Present results of any assessment of risk of bias across studies (see Item 15).	7-8, Table 2, Table S2-S5
Additional analysis	23	Give results of additional analyses, if done (e.g., sensitivity or subgroup analyses, meta-regression [see Item 16]).	N/A
**DISCUSSION**			
Summary of evidence	24	Summarize the main findings including the strength of evidence for each main outcome; consider their relevance to key groups (e.g., healthcare providers, users, and policy makers).	15-16
Limitations	25	Discuss limitations at study and outcome level (e.g., risk of bias), and at review-level (e.g., incomplete retrieval of identified research, reporting bias).	16-17
Conclusions	26	Provide a general interpretation of the results in the context of other evidence, and implications for future research.	17
**FUNDING**			
Funding	27	Describe sources of funding for the systematic review and other support (e.g., supply of data); role of funders for the systematic review.	17

Table S2. Details of meta-analyses of observational studies associating clinical intervention and all-cause mortality of patients with chronic kidney disease graded as suggestive evidence, weak evidence, or not significant

Author, year	Comparison (experimental arm vs. control arm)	CKD stages	Follow-up duration (months)* or time of outcome measurement	Number of studies	Deaths / population	Effect metrics	Summary effect estimate (95% CI) under random effects†	Summary estimate p value	I2 (%)	95% prediction interval	Evaluation of bias ‡
**Observational studies, suggestive evidence**											
Volodarskiy, et al. 2016	PCI vs. medical therapy for coronary revascularization therapy	3-5	48	15	3801 / 12647	RR	0.72 (0.6 to 0.86)	0.00039	82	0.37 to 1.39	Large heterogeneity; excess significance bias; loss of significance under 10% credibility ceiling
Khera, et al. 2018	DES vs. BMS for coronary revascularization therapy	5D	12	16	24838 / 62863	OR	0.75 (0.64 to 0.89)	0.00079	86	0.45 to 1.26	Large heterogeneity; loss of significance under 10% credibility ceiling
Lu, et al. 2016	DES vs. BMS for coronary revascularization therapy	Any	6 - 72	26	>1000 / 117247	OR	0.79 (0.71 to 0.89)	0.000042	81	0.55 to 1.15	Large heterogeneity; loss of significance under 10% credibility ceiling
Fu, et al. 2017	ICD for primary prevention of sudden cardiac death vs. no ICD	3-5	12 - 96	11	>1000 / 19808	HR	0.74 (0.63 to 0.86)	0.00013	77	0.46 to 1.18	Large heterogeneity
Apetrii, et al. 2017	Parathyroidectomy for secondary hyperparathyroidism vs. non-surgical treatment	5D	12 - 360	14	>1000 / 24003	HR	0.74 (0.66 to 0.83)	0.0000003	81	0.54 to 1.02	Large heterogeneity; small study effects
Ravani, et al. 2013	Graft as HD access vs. fistula	5HD	18	17	>1000 / 398233	RR	1.18 (1.09 to 1.27)	0.000022	81	0.92 to 1.51	Large heterogeneity
Mathew, et al. 2018	Intensive HD vs. PD	5	36 - 144	3	>1000 / 17121	HR	0.67 (0.53 to 0.84)	0.00064	91	0.04 to 11.75	Large heterogeneity; small study effects
Kelly, et al. 2017	Healthy dietary pattern low on red meat, sodium, and refined sugar vs. control	3-5	48 - 156	6	3983 / 11944	RR	0.75 (0.66 to 0.87)	0.000069	0	0.62 to 0.92	None
**Observational studies, weak evidence**											
Wang, et al. 2018	Off-pump CABG vs. on-pump CABG	3-5	Short-term mortality (in-hospital or at 30 days)	13	6110 / 196522	OR	0.82 (0.7 to 0.96)	0.016	23	0.61 to 1.1	Small study effects
Volodarskiy, et al. 2016	PCI vs. medical therapy for coronary revascularization therapy	5D	Short-term mortality (in-hospital or at 30 days)	2	418 / 2854	RR	0.6 (0.36 to 0.99)	0.046	44	NA	Loss of significance under 10% credibility ceiling
Yang, et al. 2018	Limus-eluting stent vs. paclitaxel-eluting stent for coronary revascularization therapy	3-5	12 - 26	15	732 / 6392	OR	0.78 (0.65 to 0.94)	0.0089	9	0.57 to 1.07	Loss of significance under 10% credibility ceiling
Wang, et al. 2018	DES vs. BMS for coronary revascularization therapy	5D	6 - 84	18	18763 / 44194	OR	0.78 (0.66 to 0.93)	0.0043	76	0.49 to 1.25	Large heterogeneity; loss of significance under 10% credibility ceiling
Li, et al. 2018	Combined RAAS blockade vs. ACEI or ARB	5	6 - 18	6	1226 / 12873	OR	0.71 (0.54 to 0.93)	0.012	50	0.36 to 1.39	Large heterogeneity
Crowley, et al. 2017	Metformin regimen for diabetes vs. control	3-5	12 - 47	7	NR / 33442	HR	0.78 (0.66 to 0.92)	0.0039	80	0.46 to 1.33	Large heterogeneity; loss of significance under 10% credibility ceiling
Yang, et al. 2015	Statin vs. control	5D with diabetes	24 - 52	5	NR / 13081	HR	0.81 (0.71 to 0.92)	0.0017	55	0.55 to 1.2	Large heterogeneity
Lu, et al. 2017	Vitamin D or analogues vs. non-vitamin D treatment	ND	23 - 53	4	NR / 2729	RR	0.53 (0.32 to 0.87)	0.013	76	0.06 to 4.48	Large heterogeneity; loss of significance under 10% credibility ceiling
Wongrakpanich, et al. 2017	Dialysis therapy vs. conservative management	5	12 - 216	3	357 / 1438	HR	0.53 (0.3 to 0.92)	0.023	73	0 to 281.03	Large heterogeneity; loss of significance under 10% credibility ceiling
Han, et al. 2015	HD vs. PD	5	12 - 120	15	NR / 631421	HR	0.89 (0.82 to 0.97)	0.0099	83	0.66 to 1.22	Large heterogeneity; loss of significance under 10% credibility ceiling
Shi, et al. 2018	Multidisciplinary care vs. no multidisciplinary care	Any	36	12	762 / 7390	OR	0.61 (0.43 to 0.86)	0.0052	70	0.2 to 1.91	Large heterogeneity; loss of significance under 10% credibility ceiling
**Observational studies, no association**											
Volodarskiy, et al. 2016	CABG vs. medical therapy	3-5	48	5	2335 / 6113	RR	0.76 (0.5 to 1.15)	0.19	93	0.16 to 3.6	Large heterogeneity
Volodarskiy, et al. 2016	CABG vs. medical therapy	5D	48	3	894 / 3160	RR	0.88 (0.62 to 1.26)	0.49	67	0.02 to 45.72	Large heterogeneity
Volodarskiy, et al. 2016	CABG vs. medical therapy	3-5	Short-term mortality (in-hospital or at 30 days)	3	459 / 3642	RR	1.06 (0.79 to 1.43)	0.7	0	0.15 to 7.45	None

Volodarskiy, et al. 2016	CABG vs. medical therapy	5D	Short-term mortality (in-hospital or at 30 days)	2	416 / 2645	RR	1.17 (0.82 to 1.65)	0.39	0	NA	None
Ren, et al. 2014	CABG vs. PCI	5D	12 - 60	22	48664 / 77133	OR	0.92 (0.8 to 1.06)	0.25	82	0.61 to 1.38	Large heterogeneity
Volodarskiy, et al. 2016	PCI vs. medical therapy for coronary revascularization therapy	5D	48	5	1120 / 3888	RR	0.72 (0.52 to 1)	0.051	85	0.22 to 2.4	Large heterogeneity; small study effects; excess significance bias
Lu, et al. 2016	Spironolactone vs. no mineralocorticoid receptors	Any	12 - 21	3	NR / 2863	RR	0.9 (0.71 to 1.15)	0.4	59	0.07 to 11.95	Large heterogeneity
Phan, et al. 2016	Bioprosthetic vs. mechanical valve placement	5D	19 - 120	14	NR / 6820	HR	1.2 (0.99 to 1.46)	0.068	50	0.7 to 2.07	Large heterogeneity
Cheng, et al. 2018	Trans-catheter vs. surgical aortic valve replacement	5D	Short-term mortality (in-hospital or at 30 days)	5	642 / 8064	OR	0.78 (0.51 to 1.21)	0.27	30	0.26 to 2.37	None
Cheng, et al. 2018	Trans-catheter vs. surgical aortic valve replacement	ND	Short-term mortality (in-hospital or at 30 days)	5	792 / 9619	OR	0.65 (0.41 to 1.03)	0.065	67	0.16 to 2.68	Large heterogeneity
Lei, et al. 2018	Warfarin for atrial fibrillation vs. control	5HD	13 - 120	12	9088 / 19281	OR	0.91 (0.8 to 1.03)	0.14	42	0.67 to 1.24	None
Apetrii, et al. 2017	Parathyroidectomy for secondary hyperparathyroidism vs. non-surgical treatment	5D	1	2	NR / NR	HR	1.43 (0.45 to 4.55)	0.54	97	NA	Large heterogeneity
Li, et al. 2017	Total parathyroidectomy for secondary hyperparathyroidism vs. total parathyroidectomy with autotransplantation	Any	12 - 36	4	28 / 220	RR	0.82 (0.36 to 1.86)	0.63	11	0.09 to 7.21	None
Scotland, et al. 2018	Multiple-frequency bioimpedance devices for HD fluid management vs. standard clinical assessment	Any	12	3	42 / 618	HR	0.69 (0.23 to 2.08)	0.51	54	0 to 71653.7	Large heterogeneity
Zhao, et al. 2018	Earlier PD vs. later PD	5	12 - 180	10	NR / NR	HR	1.04 (0.99 to 1.08)	0.1	49	0.94 to 1.14	None
Zhou, et al. 2018	HD vs. PD	Any with PKD	60 - 264	7	NR / 7665	RR	1.06 (0.84 to 1.34)	0.63	35	0.61 to 1.83	None

* Represented as median or range of follow-up duration of individual studies.
† Summary estimate smaller than 1 favors experimental arm (lower mortality in experimental arm); effect estimate larger than 1 favors control arm (lower mortality in control arm)
‡ Any of the following: large heterogeneity, signs of small study effects, signs of excess significance bias, or loss of statistical significance in 10% credibility ceiling.
All statistical tests are two-sided.
Abbreviations: ACEI, angiotensin-converting enzyme inhibitor; ARB, angiotensin receptor blocker; BMS, bare metal stent; CABG, coronary artery bypass; CI, confidence interval; CKD, chronic kidney disease; DES, drug-eluting stent; HD, hemodialysis; HR, hazard ratio; ICD, implantable cardioverter defibrillator; NA, not available; NR, not reported; OR, odds ratio; PCI, percutaneous intervention; PD, peritoneal dialysis; PKD, polycystic kidney disease; RAAS, renin–angiotensin–aldosterone system; RR, risk ratio; vs., versus

Table S3. Details of meta-analyses of randomized controlled trials associating clinical intervention and all-cause mortality of patients with chronic kidney disease, having p value > 0.05

Author, year	Comparison (experimental arm vs. control arm)	CKD stages	Follow-up duration (months)* or time of outcome measurement	Number of studies	Deaths / population	Effect metrics	Summary effect estimate (95% CI) under random effects†	Summary estimate p value	I2 (%)	95% prediction interval	Evaluation of bias ‡
Hahn, et al. 2017	Epoetin α for anemia treatment every 2 weeks vs. weekly	5D	1 - 14	4	22 / 838	RR	0.89 (0.38 to 2.09)	0.79	0	0.14 to 5.78	None
Palmer, et al. 2014	Epoetin β for anemia treatment vs. control therapy	Any	6	3	41 / 468	OR	0.7 (0.36 to 1.33)	0.27	0	0.01 to 46.51	None
Amato, et al. 2018	Epoetin α for anemia treatment vs. biosimilar ESA	Any	3 - 12	8	108 / 2294	RR	0.94 (0.52 to 1.69)	0.83	42	0.22 to 4.05	None
Palmer, et al. 2014	Darbepoetin α intravenous injection for anemia treatment vs. subcutaneous injection	5D	29	2	9 / 183	RR	1.29 (0.33 to 5.12)	0.72	0	NA	None
Volodarskiy, et al. 2018	1st, 2nd generation DES vs. BMS for coronary revascularization therapy	3-5	25	5	230 / 1567	RR	0.99 (0.78 to 1.27)	0.96	0	0.67 to 1.48	None
Sharma, et al. 2011	ACEI vs. placebo	3 without diabetes	36 - 42	2	170 / 1906	RR	1.79 (0.17 to 18.47)	0.62	81	NA	Large heterogeneity
Nistor, et al. 2018	ACEI or ARB single agent vs. placebo or active control	3-5ND with diabetes	29 - 48	4	828 / 5309	RR	0.97 (0.85 to 1.1)	0.6	0	0.73 to 1.28	None
Liu, et al. 2017	ACEI or ARB single agent vs. placebo or active control	5D	46	8	265 / 1746	RR	0.94 (0.75 to 1.17)	0.59	0	0.71 to 1.24	None
Zhao, et al. 2016	Calcium channel blockers vs. ACEI or ARB	Any	35 - 60	9	3566 / 25642	OR	0.96 (0.89 to 1.03)	0.21	0	0.88 to 1.04	None
Zeng, et al. 2018	Bivalirudin for coronary artery disease vs. heparin plus glycoprotein IIb/IIIa inhibitors	Any	< 1	5	147 / 3796	RR	1.12 (0.81 to 1.53)	0.5	0	0.66 to 1.87	None
Palmer, et al. 2013	Antiplatelet agent for general CKD patients vs. control therapy	3-5	1 - 60	21	1145 / 16152	RR	0.95 (0.84 to 1.08)	0.43	10	0.76 to 1.19	None
Shaw, et al. 2016	Early invasive coronary angiography and/or revascularization for non ST elevation acute coronary syndrome vs. initial conservative approach	Any	Mortality assessed in-hospital or at 6 - 12 months	5	NR / 1453	HR	0.76 (0.49 to 1.17)	0.21	14	0.3 to 1.91	None
Pun, et al. 2014	ICD for primary prevention of sudden cardiac death vs. no ICD	3b	20 - 40	3	NR / NR	HR	0.82 (0.66 to 1.01)	0.068	0	0.2 to 3.33	None
He, et al. 2018	N-acetylcysteine after cardiac surgery vs. placebo	Any	NA	5	30 / 678	RR	0.64 (0.29 to 1.4)	0.26	0	0.18 to 2.29	Small study effects
Wang, et al. 2018	Cinacalcet and/or vitamin D analogue or phosphate binders vs. placebo and/or vitamin D analogue or phosphate binders	3-5	< 12	16	NR / 8386	RR	0.97 (0.89 to 1.05)	0.42	0	0.89 to 1.06	None
Lo, et al. 2018	DPP-4 inhibitor for diabetes vs. placebo	3-5 with diabetes	NA	6	397 / 4211	RR	0.89 (0.75 to 1.06)	0.19	0	0.69 to 1.14	None
Toyama, et al. 2019	SGLT-2 inhibitor for diabetes vs. placebo	3-5 with type 2 diabetes	NA	5	593 / 7363	RR	0.86 (0.73 to 1.01)	0.069	0	0.66 to 1.12	None
Ruospo, et al. 2018	Iron-based phosphate binders vs. placebo or usual care	Any	3.7	2	3 / 239	RR	0.52 (0.06 to 4.61)	0.55	0	NA	None
Habbous, et al. 2017	Lanthanum carbonate vs. calcium-based phosphate binders	3-5	0.5 - 36	4	7 / 1564	RR	0.73 (0.18 to 3)	0.66	0	0.03 to 16.25	None
Habbous, et al. 2017	Sevelamer vs. calcium-based phosphate binders	3-5	0.5 - 36	12	751 / 5071	RR	0.62 (0.35 to 1.07)	0.085	75	0.13 to 3.03	Large heterogeneity
Sun, et al. 2015	Statin vs. placebo	5D	48 - 60	3	2900 / 7051	RR	0.98 (0.93 to 1.03)	0.41	0	0.69 to 1.39	None
Lu, et al. 2017	Vitamin D or analogues vs. non-vitamin D treatment	5D	3 - 24	9	NR / 700	RR	1.13 (0.63 to 2.03)	0.68	0	0.56 to 2.29	None
Lu, et al. 2017	Vitamin D or analogues vs. non-vitamin D treatment	ND	3 - 24	6	NR / 832	RR	1.55 (0.52 to 4.62)	0.44	0	0.33 to 7.3	None
Wang, et al. 2014	Hemodiafiltration vs. conventional HD	5	24 - 36	6	612 / 2727	RR	0.87 (0.66 to 1.16)	0.36	58	0.41 to 1.86	Large heterogeneity

Nistor, et al. 2015	Hemofiltration or hemodiafiltration or acetate-free biofiltration vs. conventional HD	5	12 - 48	11	787 / 3396	RR	0.87 (0.72 to 1.04)	0.13	33	0.58 to 1.31	None
Wang, et al. 2014	Hemofiltration vs. conventional HD	5	12 - 36	3	24 / 125	RR	0.55 (0.26 to 1.16)	0.12	0	0 to 67.71	None
Song, et al. 2010	Renal replacement therapy for prevention of acute kidney injury vs. control	3-5	NR	4	26 / 591	OR	0.36 (0.12 to 1.07)	0.067	24	0.01 to 10.41	None
Wang, et al. 2016	Citrate for alternative HD catheter lock solution vs. heparin 5000 IU/mL	5HD	6	8	63 / 1425	RR	0.88 (0.54 to 1.43)	0.6	0	0.48 to 1.61	None
McCann, et al. 2010	Topical antimicrobial ointment usage in central venous catheter HD patients vs. no ointment or placebo	5HD	6	3	28 / 322	RR	0.36 (0.12 to 1.05)	0.062	33	0 to 6751.56	None
Wang, et al. 2016	Systematic warfarin for preventing central venous HD catheter malfunction vs. placebo	5HD	6	3	26 / 403	RR	0.78 (0.37 to 1.66)	0.52	0	0.01 to 103.84	None
Htay, et al. 2018	Low glucose degradation product PD dialysate vs. standard glucose dialysate	5PD	1-24	13	80 / 1229	RR	0.74 (0.47 to 1.14)	0.17	0	0.45 to 1.21	None
Htay, et al. 2018	Glucose polymer PD dialysate vs. standard glucose diasylate	5PD	1-24	5	16 / 816	RR	0.82 (0.42 to 1.59)	0.55	0	0.28 to 2.42	None
Xie, et al. 2011	Coiled intraperitoneal segment PD catheters vs. straight intraperitoneal segment catheters	5PD	12 - 32	4	48 / 317	RR	0.94 (0.56 to 1.57)	0.81	0	0.3 to 2.9	None
Sampson, et al. 2017	Allopurinol as uric acid lowering therapy vs. usual care	Any	24	2	7 / 218	RR	0.13 (0.02 to 1.06)	0.056	0	NA	None
Jun, et al. 2012	Antioxidants vs. control	3-5	12 - 48	5	299 / 1727	RR	0.93 (0.76 to 1.14)	0.46	0	0.67 to 1.29	None
Shi, et al. 2018	Multidisciplinary care vs. no multidisciplinary care	Any	36	4	240 / 1912	OR	0.82 (0.53 to 1.27)	0.39	41	0.18 to 3.8	None
Valentijn, et al. 2018	Person-centered integrated care vs. control	Any	12	11	270 / 4126	RR	0.86 (0.68 to 1.09)	0.21	6	0.6 to 1.23	None
Silver, et al. 2017	Quality improvement strategy vs. usual care	3-5	12	8	333 / 3853	RR	0.94 (0.72 to 1.23)	0.65	21	0.55 to 1.6	None
Palmer, et al. 2017	Dietary counselling vs. control	3-5	12	4	18 / 371	RR	1.58 (0.6 to 4.18)	0.36	0	0.19 to 13.33	None
Jun, et al. 2012	Fibrate vs. placebo	3	61	2	128 / 918	RR	0.86 (0.63 to 1.19)	0.37	0	NA	None
Jun, et al. 2012	Fibrate vs. placebo	1-2	61	2	969 / 11408	RR	1.01 (0.8 to 1.27)	0.94	74	NA	Large heterogeneity
Nigwekar, et al. 2016	Folic acid and/or vitamin B6 and/or vitamin B12 vs. control	5D	23 - 43	6	819 / 2447	RR	1 (0.9 to 1.12)	1	0	0.85 to 1.17	None
Jardine, et al. 2012	Folic acid and/or vitamin B6 and/or vitamin B12 vs. control	3-5	32 - 60	4	756 / 2215	RR	1.04 (0.93 to 1.16)	0.45	0	0.82 to 1.33	None
Hahn, et al. 2018	Low protein diet vs. normal protein diet	3-5ND	12 - 50	5	48 / 1680	RR	0.78 (0.51 to 1.19)	0.25	0	0.39 to 1.55	None

* Represented as median or range of follow-up duration of individual studies.
† Summary estimate smaller than 1 favors experimental arm (lower mortality in experimental arm); effect estimate larger than 1 favors control arm (lower mortality in control arm)
‡ Any of the following: large heterogeneity, signs of small study effects, or signs of excess significance bias.
All statistical tests are two-sided.
Abbreviations: ACEI, angiotensin-converting enzyme inhibitor; ARB, angiotensin receptor blocker; BMS, bare metal stent; CI, confidence interval; CKD, chronic kidney disease; DES, drug-eluting stent; DPP-4, Dipeptidylpeptidase-4; ESA, erythropoiesis-stimulating agent; HD, hemodialysis; HR, hazard ratio; ICD, implantable cardioverter defibrillator; NA, not available; NR, not reported; OR, odds ratio; PD, peritoneal dialysis; RR, risk ratio; SGLT-2, sodium glucose cotransporter-2; vs., versus

Table S4. Details of credibility assessment in meta-analyses of observational studies associating clinical intervention and all-cause mortality of patients with chronic kidney disease

Author, year	Comparison (experimental arm vs. control arm)	CKD stage	Effect metrics	Fixed effects summary estimate*	Fixed effects p value	Largest study summary estimate (95% CI)*	Egger p value	Excess significance test p value	Random effects summary esimate (95% CI) under 5%/10%/15%/20% credibility ceiling*	I2 (%) under random effects/5%/10%/15%/20% credibility ceiling
Wang, et al. 2018	Off-pump CABG vs. on-pump CABG	3-5	OR	0.88 (0.83 to 0.94)	0.00016	0.9 (0.84 to 0.97)	0.0029	The expected number of significant studies was larger than the observed number of significant studies	0.87 (0.79 to 0.96) / 0.87 (0.76 to 0.98) / 0.86 (0.74 to 1.01) / 0.85 (0.71 to 1.03)	23/0/0/0/0
Volodarskiy, et al. 2016	CABG vs. medical therapy	3-5	RR	0.91 (0.84 to 0.99)	0.035	1.07 (0.97 to 1.18)	0.37	Random effect summary estimate was not significant	0.87 (0.65 to 1.18) / 0.96 (0.73 to 1.24) / 1.03 (0.84 to 1.25) / 1.06 (0.91 to 1.22)	93/62/42/13/0
Volodarskiy, et al. 2016	CABG vs. medical therapy	5D	RR	0.89 (0.76 to 1.04)	0.15	0.87 (0.73 to 1.04)	0.93	Random effect summary estimate was not significant	0.91 (0.65 to 1.28) / 0.94 (0.68 to 1.31) / 0.95 (0.7 to 1.31) / 0.94 (0.72 to 1.23)	67/59/50/31/0
Volodarskiy, et al. 2016	CABG vs. medical therapy	3-5	RR	1.06 (0.79 to 1.43)	0.7	1.26 (0.86 to 1.85)	0.3	Random effect summary estimate was not significant	1.06 (0.79 to 1.43) / 1.06 (0.79 to 1.43) / 1.03 (0.75 to 1.43) / 0.99 (0.69 to 1.41)	0/0/0/0/0
Volodarskiy, et al. 2016	CABG vs. medical therapy	5D	RR	1.17 (0.82 to 1.65)	0.39	1.26 (0.86 to 1.85)	NA	Random effect summary estimate was not significant	1.17 (0.82 to 1.65) / 1.17 (0.82 to 1.65) / 1.15 (0.78 to 1.68) / 1.11 (0.71 to 1.74)	0/0/0/0/0
Kannan, et al. 2016	CABG vs. PCI	<5	OR	0.82 (0.76 to 0.88)	0.00000028	0.81 (0.75 to 0.88)	0.67	The expected number of significant studies was larger than the observed number of significant studies	0.85 (0.7 to 1.03) / 0.87 (0.69 to 1.08) / 0.88 (0.69 to 1.12) / 0.9 (0.69 to 1.18)	0/0/0/0/0
Ren, et al. 2014	CABG vs. PCI	5D	OR	0.87 (0.84 to 0.89)	5.7E-20	0.81 (0.76 to 0.85)	0.59	Random effect summary estimate was not significant	0.93 (0.81 to 1.08) / 0.96 (0.85 to 1.08) / 0.99 (0.89 to 1.11) / 1 (0.87 to 1.14)	82/33/8/0/0
Volodarskiy, et al. 2016	CABG vs. PCI	5D	RR	2.31 (2.15 to 2.48)	4.4E-119	2.41 (2.15 to 2.71)	0.88	The expected number of significant studies was larger than the observed number of significant studies	1.89 (1.3 to 2.77) / 1.66 (1.1 to 2.5) / 1.48 (0.93 to 2.37) / 1.48 (0.84 to 2.61)	40/9/0/0/0
Volodarskiy, et al. 2016	CABG vs. PCI	3-5	RR	2.21 (2.06 to 2.37)	8.1E-112	2.41 (2.15 to 2.71)	0.19	The expected number of significant studies was larger than the observed number of significant studies	1.44 (1 to 2.06) / 1.22 (0.88 to 1.71) / 1.03 (0.76 to 1.37) / 1 (0.72 to 1.4)	75/42/22/0/0
Volodarskiy, et al. 2016	PCI vs. medical therapy for coronary revascularization therapy	5D	RR	0.86 (0.77 to 0.96)	0.0085	1.11 (0.95 to 1.29)	0.042	Random effect summary estimate was not significant	0.79 (0.58 to 1.08) / 0.85 (0.64 to 1.13) / 0.91 (0.71 to 1.17) / 0.99 (0.8 to 1.21)	85/64/49/25/0
Volodarskiy, et al. 2016	PCI vs. medical therapy for coronary revascularization therapy	3-5	RR	0.79 (0.74 to 0.84)	3.3E-13	1.06 (0.96 to 1.18)	0.26	0.00048	0.8 (0.67 to 0.94) / 0.86 (0.73 to 1.01) / 0.99 (0.9 to 1.09) / 0.99 (0.88 to 1.11)	82/47/26/1/0
Volodarskiy, et al. 2016	PCI vs. medical therapy for coronary revascularization therapy	3-5	RR	0.84 (0.72 to 0.97)	0.018	0.9 (0.74 to 1.09)	0.89	Random effect summary estimate was not significant	0.88 (0.68 to 1.14) / 0.9 (0.76 to 1.06) / 0.9 (0.76 to 1.07) / 0.9 (0.73 to 1.1)	66/36/0/0/0
Volodarskiy, et al. 2016	PCI vs. medical therapy for coronary revascularization therapy	5D	RR	0.57 (0.4 to 0.81)	0.0019	0.49 (0.32 to 0.75)	NA	The expected number of significant studies was larger than the observed number of significant studies	0.68 (0.41 to 1.15) / 0.72 (0.41 to 1.26) / 0.75 (0.42 to 1.35) / 0.77 (0.42 to 1.42)	44/0/0/0/0
Yang, et al. 2018	Limus-eluting stent vs. paclitaxel-eluting stent for coronary revascularization therapy	3-5	OR	0.79 (0.67 to 0.94)	0.0065	0.73 (0.52 to 1.03)	0.14	Unobtainable because necessary data was not reported	0.82 (0.69 to 0.98) / 0.87 (0.72 to 1.05) / 0.9 (0.74 to 1.1) / 0.93 (0.75 to 1.14)	9/0/0/0/0
Lu, et al. 2016	DES vs. BMS for coronary revascularization therapy	Any	OR	0.86 (0.84 to 0.89)	1.8E-26	0.85 (0.82 to 0.89)	0.21	Unobtainable because necessary data was not reported	0.9 (0.8 to 1.01) / 0.96 (0.87 to 1.06) / 1.01 (0.93 to 1.09) / 1.01 (0.92 to 1.11)	81/43/14/0/0
Khera, et al. 2018	DES vs. BMS for coronary revascularization therapy	5D	OR	0.82 (0.79 to 0.85)	7.7E-27	0.74 (0.71 to 0.78)	0.54	Unobtainable because necessary data was not reported	0.84 (0.73 to 0.97) / 0.92 (0.83 to 1.03) / 0.97 (0.89 to 1.06) / 0.97 (0.87 to 1.08)	86/39/9/0/0
Wang, et al. 2018	DES vs. BMS for coronary revascularization therapy	5D	OR	0.88 (0.85 to 0.92)	2.2E-09	0.81 (0.77 to 0.86)	0.32	Unobtainable because necessary data was not reported	0.86 (0.74 to 1) / 1 (0.92 to 1.08) / 1 (0.91 to 1.1) / 0.99 (0.89 to 1.12)	76/25/0/0/0
Qin, et al. 2016	ACEI or ARB vs. no ACEI or ARB	ND	HR	0.82 (0.8 to 0.84)	3.4E-42	0.81 (0.78 to 0.84)	0.74	The expected number of significant studies was larger than the observed number of significant studies	0.87 (0.82 to 0.93) / 0.89 (0.82 to 0.96) / 0.9 (0.83 to 0.98) / 0.91 (0.83 to 1)	44/0/0/0/0
Li, et al. 2018	Combined RAAS blockade vs. ACEI or ARB	5	OR	0.77 (0.67 to 0.88)	0.000084	0.77 (0.64 to 0.92)	0.36	The expected number of significant studies was larger than the observed number of significant studies	0.69 (0.5 to 0.96) / 0.68 (0.48 to 0.97) / 0.67 (0.46 to 0.98) / 0.66 (0.44 to 0.99)	50/50/50/48/44

Lu, et al. 2016	Spironolactone vs. no mineralocorticoid receptors	Any	RR	0.9 (0.78 to 1.05)	0.19	1.05 (0.84 to 1.31)	0.99	Random effect summary estimate was not significant	0.94 (0.77 to 1.16) / 0.98 (0.82 to 1.17) / 1 (0.83 to 1.19) / 1.01 (0.84 to 1.21)	59/25/0/0/0
Shaw, et al. 2016	Early invasive coronary angiography and/or revascularization for non ST elevation acute coronary syndrome vs. Initial conservative approach	Any	HR	0.5 (0.47 to 0.53)	8.2E-104	0.54 (0.49 to 0.6)	0.91	Unobtainable because necessary data was not reported	0.57 (0.45 to 0.72) / 0.57 (0.43 to 0.77) / 0.57 (0.4 to 0.83) / 0.57 (0.36 to 0.9)	79/0/0/0/0
Shurrab, et al. 2018	ICD for primary prevention of sudden cardiac death vs. no ICD	5D	OR	0.47 (0.39 to 0.56)	1.3E-17	0.44 (0.36 to 0.53)	0.3	The expected number of significant studies was larger than the observed number of significant studies	0.59 (0.39 to 0.89) / 0.64 (0.4 to 1.02) / 0.68 (0.4 to 1.15) / 0.72 (0.41 to 1.27)	17/0/0/0/0
Fu, et al. 2017	ICD for primary prevention of sudden cardiac death vs. no ICD	3-5	HR	0.8 (0.76 to 0.84)	9.9E-21	0.86 (0.81 to 0.91)	0.28	Unobtainable because necessary data was not reported	0.82 (0.72 to 0.92) / 0.85 (0.75 to 0.96) / 0.87 (0.75 to 1) / 0.88 (0.76 to 1.03)	77/11/0/0/0
Phan, et al. 2016	Bioprosthetic vs. mechanical valve placement	5D	HR	1.06 (1 to 1.12)	0.047	1.04 (0.98 to 1.1)	0.21	Random effect summary estimate was not significant	1.08 (0.95 to 1.24) / 1.04 (0.99 to 1.11) / 1.04 (0.97 to 1.12) / 1.04 (0.96 to 1.13)	50/12/0/0/0
Cheng, et al. 2018	Trans-catheter vs. surgical aortic valve replacement	5D	OR	0.78 (0.55 to 1.11)	0.17	1.37 (0.68 to 2.77)	0.86	Random effect summary estimate was not significant	0.86 (0.59 to 1.26) / 0.9 (0.61 to 1.33) / 0.92 (0.62 to 1.36) / 0.92 (0.62 to 1.38)	30/0/0/0/0
Cheng, et al. 2018	Trans-catheter vs. surgical aortic valve replacement	ND	OR	0.51 (0.43 to 0.61)	1.2E-14	0.45 (0.37 to 0.55)	0.2	Random effect summary estimate was not significant	0.71 (0.43 to 1.18) / 0.68 (0.44 to 1.04) / 0.66 (0.4 to 1.09) / 0.66 (0.36 to 1.22)	67/38/2/0/0
Dahal, et al. 2016	Warfarin for atrial fibrillation vs. no warfarin	ND	HR	0.64 (0.61 to 0.68)	2.5E-57	0.63 (0.59 to 0.67)	0.98	The expected number of significant studies was larger than the observed number of significant studies	0.7 (0.56 to 0.88) / 0.7 (0.52 to 0.94) / 0.7 (0.49 to 1.01) / 0.7 (0.45 to 1.1)	39/0/0/0/0
Lei, et al. 2018	Warfarin for atrial fibrillation vs. control	5HD	OR	0.95 (0.89 to 1.02)	0.17	1.05 (0.95 to 1.16)	0.48	Random effect summary estimate was not significant	0.94 (0.84 to 1.04) / 0.99 (0.91 to 1.07) / 0.99 (0.92 to 1.08) / 0.99 (0.91 to 1.08)	42/20/0/0/0
Crowley, et al. 2017	Metformin regimen for diabetes vs. control	3-5	HR	0.79 (0.74 to 0.84)	2.2E-12	0.87 (0.77 to 0.99)	0.93	Unobtainable because necessary data was not reported	0.87 (0.78 to 0.98) / 0.9 (0.8 to 1.01) / 0.92 (0.81 to 1.05) / 0.94 (0.81 to 1.08)	80/13/0/0/0
Apetrii, et al. 2017	Parathyroidectomy for secondary hyperparathyroidism vs. non-surgical treatment	5D	HR	1.15 (0.97 to 1.36)	0.11	0.8 (0.65 to 0.98)	NA	Random effect summary estimate was not significant	1.26 (0.41 to 3.88) / 1.15 (0.4 to 3.36) / 1.03 (0.4 to 2.63) / 0.86 (0.49 to 1.5)	97/74/58/35/2
Apetrii, et al. 2017	Parathyroidectomy for secondary hyperparathyroidism vs. non-surgical treatment	5D	HR	0.92 (0.89 to 0.94)	1.9E-09	0.96 (0.92 to 1)	0.000 14	Unobtainable because necessary data was not reported	0.84 (0.76 to 0.94) / 0.9 (0.83 to 0.98) / 0.93 (0.88 to 1) / 0.93 (0.86 to 1.01)	81/46/13/0/0
Li, et al. 2017	Total parathyroidectomy for secondary hyperparathyroidism vs. total parathyroidectomy with autotransplantation	Any	RR	0.82 (0.39 to 1.75)	0.61	0.66 (0.22 to 1.96)	0.55	Random effect summary estimate was not significant	0.82 (0.36 to 1.86) / 0.82 (0.36 to 1.86) / 0.89 (0.41 to 1.93) / 0.92 (0.42 to 1.99)	11/11/11/0/0
Yang, et al. 2015	Statin vs. control	5D with diabete s	HR	0.83 (0.78 to 0.88)	0.000000 013	0.82 (0.76 to 0.89)	0.37	Unobtainable because necessary data was not reported	0.86 (0.78 to 0.96) / 0.88 (0.79 to 0.98) / 0.88 (0.79 to 0.99) / 0.89 (0.79 to 1)	55/2/0/0/0
Lu, et al. 2017	Vitamin D or analogues vs. non-vitamin D treatment	5D	RR	0.83 (0.81 to 0.85)	4.1E-57	0.89 (0.86 to 0.93)	0.019	Unobtainable because necessary data was not reported	0.75 (0.65 to 0.86) / 0.81 (0.71 to 0.92) / 0.96 (0.92 to 1.01) / 0.97 (0.92 to 1.01)	94/54/27/0/0
Lu, et al. 2017	Vitamin D or analogues vs. non-vitamin D treatment	ND	RR	0.61 (0.49 to 0.75)	0.000002 5	0.76 (0.58 to 0.99)	0.46	Unobtainable because necessary data was not reported	0.72 (0.54 to 0.94) / 0.73 (0.52 to 1.01) / 0.74 (0.5 to 1.07) / 0.75 (0.49 to 1.14)	76/0/0/0/0
Scotland, et al. 2018	Multiple-frequency bioimpedance devices for HD fluid management vs. standard clinical assessment	Any	HR	0.83 (0.41 to 1.68)	0.61	1.33 (0.48 to 3.68)	0.12	Random effect summary estimate was not significant	0.82 (0.32 to 2.12) / 0.97 (0.47 to 2.01) / 1 (0.48 to 2.08) / 1.03 (0.49 to 2.14)	54/34/1/0/0
Wongrakpanich, et al. 2017	Dialysis therapy vs. conservative management	5	HR	0.58 (0.44 to 0.75)	0.000048	0.46 (0.31 to 0.68)	0.6	The expected number of significant studies was larger than the observed number of significant studies	0.63 (0.36 to 1.11) / 0.77 (0.53 to 1.11) / 0.79 (0.54 to 1.16) / 0.81 (0.55 to 1.19)	73/33/0/0/0
Zhao, et al. 2018	Earlier HD vs. later HD	5	HR	1.05 (1.04 to 1.06)	2.3E-37	1.04 (1.03 to 1.05)	0.078	Unobtainable because necessary data was not reported	1.05 (1.01 to 1.1) / 1.03 (1 to 1.06) / 1.03 (1 to 1.07) / 1.03 (0.99 to 1.07)	98/25/0/0/0
Zhao, et al. 2018	Earlier PD vs. later PD	5	HR	1.03 (1.01 to 1.05)	0.0003	1.03 (1.01 to 1.06)	0.54	Random effect summary estimate was not significant	1.03 (0.99 to 1.08) / 1.03 (1 to 1.06) / 1.03 (1 to 1.07) / 1.03 (0.99 to 1.08)	49/26/0/0/0

Ravani, et al. 2013	Catheter as HD access vs. fistula	5HD	RR	1.41 (1.38 to 1.45)	1.1E-161	1.3 (1.25 to 1.35)	0.085	Unobtainable because necessary data was not reported	1.33 (1.2 to 1.47) / 1.28 (1.14 to 1.44) / 1.23 (1.08 to 1.41) / 1.19 (1.03 to 1.37)	83/0/0/0/0
Ravani, et al. 2013	Catheter as HD access vs. graft	5HD	RR	1.36 (1.33 to 1.39)	8.8E-126	1.46 (1.41 to 1.51)	0.97	Unobtainable because necessary data was not reported	1.19 (1.1 to 1.28) / 1.17 (1.07 to 1.28) / 1.16 (1.05 to 1.28) / 1.16 (1.03 to 1.3)	85/0/0/0/0
Ravani, et al. 2013	Graft as HD access vs. fistula	5HD	RR	1.17 (1.14 to 1.2)	1.5E-38	1.05 (1 to 1.1)	0.71	Unobtainable because necessary data was not reported	1.08 (1.04 to 1.13) / 1.08 (1.03 to 1.14) / 1.08 (1.01 to 1.14) / 1.07 (1 to 1.15)	81/0/0/0/0
Zhou, et al. 2018	HD vs. PD	Any with PKD	RR	1.16 (0.99 to 1.35)	0.064	1.4 (1.13 to 1.74)	0.084	Random effect summary estimate was not significant	1.04 (0.86 to 1.26) / 1.01 (0.82 to 1.23) / 1 (0.81 to 1.23) / 1 (0.8 to 1.24)	35/0/0/0/0
Han, et al. 2015	HD vs. PD	5	HR	0.91 (0.89 to 0.94)	2.1E-09	0.83 (0.79 to 0.88)	0.54	Unobtainable because necessary data was not reported	0.92 (0.85 to 1) / 0.94 (0.87 to 1.01) / 0.94 (0.88 to 1.01) / 0.95 (0.87 to 1.02)	83/50/22/0/0
Mathew, et al. 2018	Intensive HD vs. PD	5	HR	0.74 (0.69 to 0.79)	1.2E-19	0.8 (0.73 to 0.88)	0.074	Unobtainable because necessary data was not reported	0.76 (0.62 to 0.93) / 0.76 (0.58 to 0.99) / 0.76 (0.55 to 1.05) / 0.76 (0.51 to 1.14)	91/0/0/0/0
Jin, et al. 2013	Prolonged nocturnal or daytime HD vs. conventional HD	5	OR	0.81 (0.77 to 0.85)	2E-15	0.9 (0.83 to 0.98)	0.0018	Unobtainable because necessary data was not reported	0.84 (0.77 to 0.91) / 0.84 (0.75 to 0.93) / 0.84 (0.74 to 0.95) / 0.84 (0.72 to 0.98)	68/0/0/0/0
Shi, et al. 2018	Multidisciplinary care vs. no multidisciplinary care	Any	OR	0.68 (0.57 to 0.81)	0.000021	0.8 (0.53 to 1.21)	0.17	0.19	0.74 (0.55 to 0.99) / 0.8 (0.61 to 1.05) / 0.84 (0.66 to 1.07) / 0.83 (0.62 to 1.11)	70/44/21/0/0
Smart, et al. 2014	Early referral to specialist nephrology services vs. late referral to specialist nephrology services	Any	RR	0.67 (0.64 to 0.71)	6.4E-47	0.83 (0.77 to 0.9)	0.035	The expected number of significant studies was larger than the observed number of significant studies	0.7 (0.61 to 0.82) / 0.7 (0.59 to 0.84) / 0.71 (0.57 to 0.88) / 0.72 (0.56 to 0.93)	82/0/0/0/0
Kelly, et al. 2017	Healthy dietary pattern low on red meat, sodium, and refined sugar vs. control	3-5	RR	0.75 (0.66 to 0.87)	0.000069	0.77 (0.61 to 0.97)	0.61	The expected number of significant studies was larger than the observed number of significant studies	0.79 (0.67 to 0.94) / 0.81 (0.67 to 0.98) / 0.83 (0.68 to 1.02) / 0.85 (0.68 to 1.06)	0/0/0/0/0
Remschmidt, et al. 2014	Influenza vaccine vs. control	5	OR	0.71 (0.7 to 0.72)	0	0.71 (0.7 to 0.72)	0.64	Unobtainable because necessary data was not reported	0.73 (0.59 to 0.89) / 0.73 (0.56 to 0.94) / 0.73 (0.53 to 1) / 0.73 (0.49 to 1.07)	83/0/0/0/0

* Effect estimate smaller than 1 favors experimental arm (lower mortality in experimental arm); effect estimate larger than 1 favors control arm (lower mortality in control arm)
All statistical tests are two-sided.
Abbreviations: ACEI, angiotensin-converting enzyme inhibitor; ARB, angiotensin receptor blocker; BMS, bare metal stent; CABG, coronary artery bypass; CI, confidence interval; CKD, chronic kidney disease; DES, drug-eluting stent; HD, hemodialysis; HR, hazard ratio; ICD, implantable cardioverter defibrillator; NA, not available; OR, odds ratio; PCI, percutaneous intervention; PD, peritoneal dialysis; RAAS, renin–angiotensin–aldosterone system; RR, risk ratio; vs., versus

Table S5. Details of credibility assessment in meta-analyses of randomized controlled trials associating clinical intervention and all-cause mortality of patients with chronic kidney disease

Author, year	Comparison (experimental arm vs. control arm)	CKD stage	Effect metrics	Fixed effects summary estimate*	Fixed effects p value	Largest study summary estimate (95% CI)*	Egger p value	Excess significance test p value
Hahn, et al. 2017	Epoetin α for anemia treatment every 2 weeks vs. weekly	5D	RR	0.89 (0.38 to 2.09)	0.79	0.67 (0.19 to 2.34)	0.0098	Random effect summary estimate was not significant
Palmer, et al. 2014	Epoetin β for anemia treatment vs. control therapy	Any	OR	0.7 (0.36 to 1.33)	0.27	0.76 (0.37 to 1.57)	0.19	Random effect summary estimate was not significant
Amato, et al. 2018	Epoetin α for anemia treatment vs. biosimilar ESA	Any	RR	0.93 (0.62 to 1.38)	0.72	1.23 (0.6 to 2.52)	0.69	Random effect summary estimate was not significant
Palmer, et al. 2014	Darbepoetin α intravenous injection for anemia treatment vs. subcutaneous injection	5D	RR	1.29 (0.33 to 5.12)	0.72	0.77 (0.13 to 4.49)	NA	Random effect summary estimate was not significant
Palmer, et al. 2010	High vs. low hemoglobin target for anemia treatment	2-5	RR	1.09 (0.99 to 1.2)	0.072	1.05 (0.93 to 1.19)	0.13	Random effect summary estimate was not significant
Ye, et al. 2018	High vs. low hemoglobin target for anemia treatment	5D	RR	1.11 (0.95 to 1.3)	0.19	1.21 (1.01 to 1.44)	0.19	Random effect summary estimate was not significant
Volodarskiy, et al. 2018	1st, 2nd generation DES vs. BMS for coronary revascularization therapy	3-5	RR	0.99 (0.78 to 1.27)	0.96	1.03 (0.72 to 1.47)	0.66	Random effect summary estimate was not significant
Sharma, et al. 2011	ACEI vs. placebo	3 without diabetes	RR	0.7 (0.53 to 0.93)	0.015	0.67 (0.5 to 0.89)	NA	Random effect summary estimate was not significant
Liu, et al. 2017	ACEI or ARB single agent vs. placebo or active control	5D	RR	0.94 (0.75 to 1.17)	0.59	1.09 (0.78 to 1.52)	0.54	Random effect summary estimate was not significant
Nistor, et al. 2018	ACEI or ARB single agent vs. placebo or active control	3-5ND with diabetes	RR	0.97 (0.85 to 1.1)	0.6	1.03 (0.85 to 1.25)	0.84	Random effect summary estimate was not significant
Badve, et al. 2011	Beta-blockers for heart failure vs. placebo	3-5	RR	0.72 (0.64 to 0.8)	2.6E-09	0.76 (0.64 to 0.91)	0.33	The expected number of significant studies was larger than the observed number of significant studies
Heerspink, et al. 2009	More intensive vs. less intensive blood pressure target	5D	RR	0.8 (0.71 to 0.91)	0.00038	0.8 (0.68 to 0.94)	0.53	The expected number of significant studies was larger than the observed number of significant studies
Malhotra, et al. 2017	More intensive vs. less intensive blood pressure target	3-5ND	OR	0.86 (0.76 to 0.96)	0.01	0.86 (0.66 to 1.12)	0.081	The expected number of significant studies was larger than the observed number of significant studies
Zhao, et al. 2016	Calcium channel blockers vs. ACEI or ARB	Any	OR	0.96 (0.89 to 1.03)	0.21	0.97 (0.88 to 1.07)	0.22	Random effect summary estimate was not significant
Lu, et al. 2016	Spironolactone or eplerenone vs. no mineralocorticoid receptors	Any	RR	0.72 (0.58 to 0.91)	0.0048	0.87 (0.67 to 1.13)	0.16	Unobtainable because necessary data was not reported
Quach, et al. 2016	Spironolactone or eplerenone vs. placebo or none	5	RR	0.4 (0.23 to 0.7)	0.0012	0.32 (0.16 to 0.64)	0.76	The expected number of significant studies was larger than the observed number of significant studies
Zeng, et al. 2018	Bivalirudin for coronary artery disease vs. heparin plus glycoprotein IIb/IIIa inhibitors	Any	RR	1.12 (0.81 to 1.53)	0.5	1.07 (0.61 to 1.87)	0.42	Random effect summary estimate was not significant
Palmer, et al. 2013	Antiplatelet agent for general CKD patients vs. control therapy	3-5	RR	0.96 (0.87 to 1.07)	0.49	0.93 (0.75 to 1.16)	0.091	Random effect summary estimate was not significant
Shaw, et al. 2016	Early invasive coronary angiography and/or revascularization for non ST elevation acute coronary syndrome vs. initial conservative approach	Any	HR	0.76 (0.51 to 1.12)	0.17	0.67 (0.32 to 1.4)	0.78	Random effect summary estimate was not significant
Pun, et al. 2014	ICD for primary prevention of sudden cardiac death vs. no ICD	3b	HR	0.82 (0.66 to 1.01)	0.068	0.93 (0.68 to 1.27)	0.4	Random effect summary estimate was not significant
Pun, et al. 2014	ICD for primary prevention of sudden cardiac death vs. no ICD	1	HR	0.48 (0.34 to 0.67)	0.000017	0.55 (0.33 to 0.9)	0.16	Unobtainable because necessary data was not reported
He, et al. 2018	N-acetylcysteine after cardiac surgery vs. placebo	Any	RR	0.64 (0.29 to 1.4)	0.26	1.21 (0.33 to 4.42)	0.045	Random effect summary estimate was not significant
Wang, et al. 2018	Cinacalcet and/or vitamin D analogue or phosphate binders vs. placebo and/or vitamin D analogue or phosphate binders	3-5	RR	0.97 (0.89 to 1.05)	0.42	0.97 (0.89 to 1.05)	0.28	Random effect summary estimate was not significant
Lo, et al. 2018	DPP-4 inhibitor for diabetes vs. placebo	3-5 with diabetes	RR	0.89 (0.75 to 1.06)	0.19	0.88 (0.74 to 1.05)	0.37	Random effect summary estimate was not significant
Toyama, et al. 2019	SGLT-2 inhibitor for diabetes vs. placebo	3-5 with type 2 diabetes	RR	0.86 (0.73 to 1.01)	0.069	0.88 (0.67 to 1.15)	0.15	Random effect summary estimate was not significant
Ruospo, et al. 2018	Iron-based phosphate binders vs. placebo or usual care	Any	RR	0.52 (0.06 to 4.61)	0.55	0.2 (0.01 to 4.02)	NA	Random effect summary estimate was not significant
Habbous, et al. 2017	Lanthanum carbonate vs. calcium-based phosphate binders	3-5	RR	0.73 (0.18 to 3)	0.66	0.52 (0.05 to 5.38)	0.93	Random effect summary estimate was not significant

Wang, et al. 2018	Lanthanum carbonate vs. calcium-based phosphate binders or sevelamer	5HD	OR	0.45 (0.32 to 0.63)	0.0000029	0.4 (0.27 to 0.59)	0.11	The expected number of significant studies was larger than the observed number of significant studies
Sekercioglu, et al. 2016	Non-calcium-based phosphate binders vs. calcium-based phosphate binders	3-5	RR	0.84 (0.75 to 0.93)	0.0015	0.97 (0.84 to 1.12)	0.14	Unobtainable because necessary data was not reported
Habbous, et al. 2017	Sevelamer vs. calcium-based phosphate binders	3-5	RR	0.82 (0.71 to 0.93)	0.0022	0.98 (0.85 to 1.13)	0.35	Random effect summary estimate was not significant
Zhang, et al. 2014	Statin vs. less statin or placebo	ND	RR	0.79 (0.73 to 0.85)	1.2E-09	0.8 (0.73 to 0.88)	0.54	The expected number of significant studies was larger than the observed number of significant studies
Sun, et al. 2015	Statin vs. placebo	5D	RR	0.98 (0.93 to 1.03)	0.41	0.96 (0.89 to 1.04)	0.85	Random effect summary estimate was not significant
Lu, et al. 2017	Vitamin D or analogues vs. non-vitamin D treatment	5D	RR	1.13 (0.63 to 2.03)	0.68	1.15 (0.41 to 3.21)	0.082	Random effect summary estimate was not significant
Lu, et al. 2017	Vitamin D or analogues vs. non-vitamin D treatment	ND	RR	1.55 (0.52 to 4.62)	0.44	3.91 (0.45 to 34.13)	0.06	Random effect summary estimate was not significant
Wang, et al. 2014	Hemodiafiltration vs. conventional HD	5	RR	0.85 (0.74 to 0.97)	0.017	0.94 (0.78 to 1.14)	0.77	Random effect summary estimate was not significant
Wang, et al. 2014	Hemofiltration vs. conventional HD	5	RR	0.55 (0.26 to 1.16)	0.12	0.58 (0.26 to 1.29)	0.77	Random effect summary estimate was not significant
Nistor, et al. 2015	Hemofiltration or hemodiafiltration or acetate-free biofiltration vs. conventional HD	5	RR	0.87 (0.77 to 0.98)	0.022	0.94 (0.78 to 1.14)	0.91	Random effect summary estimate was not significant
Song, et al. 2010	Renal replacement therapy for prevention of acute kidney injury vs. control	3-5	OR	0.36 (0.14 to 0.9)	0.029	0.2 (0.05 to 0.88)	0.85	Random effect summary estimate was not significant
Wang, et al. 2016	Citrate for alternative HD catheter lock solution vs. heparin 5000 IU/mL	5HD	RR	0.88 (0.54 to 1.43)	0.6	1.4 (0.61 to 3.21)	0.94	Random effect summary estimate was not significant
McCann, et al. 2010	Topical antimicrobial ointment usage in central venous catheter HD patients vs. no ointment or placebo	5HD	RR	0.37 (0.16 to 0.85)	0.019	0.22 (0.07 to 0.72)	0.74	Random effect summary estimate was not significant
Wang, et al. 2016	Systematic warfarin for preventing central venous HD catheter malfunction vs. placebo	5HD	RR	0.78 (0.37 to 1.66)	0.52	0.63 (0.21 to 1.86)	0.67	Random effect summary estimate was not significant
Tan, et al. 2018	High-flux HD vs. low-flux HD	5	RR	0.71 (0.63 to 0.8)	8.5E-09	0.68 (0.53 to 0.87)	0.65	The expected number of significant studies was larger than the observed number of significant studies
Htay, et al. 2018	Low glucose degradation product PD dialysate vs. standard glucose dialysate	5PD	RR	0.74 (0.47 to 1.14)	0.17	1.14 (0.46 to 2.82)	0.51	Random effect summary estimate was not significant
Htay, et al. 2018	Glucose polymer PD dialysate vs. standard glucose dialysate	5PD	RR	0.82 (0.42 to 1.59)	0.55	0.82 (0.32 to 2.12)	0.66	Random effect summary estimate was not significant
Xie, et al. 2011	Coiled intraperitoneal segment PD catheters vs. straight intraperitoneal segment catheters	5PD	RR	0.94 (0.56 to 1.57)	0.81	0.91 (0.44 to 1.89)	0.21	Random effect summary estimate was not significant
Sampson, et al. 2017	Allopurinol as uric acid lowering therapy vs. usual care	Any	RR	0.13 (0.02 to 1.06)	0.056	0.09 (0.01 to 1.61)	NA	Random effect summary estimate was not significant
Jun, et al. 2012	Antioxidants vs. control	3-5	RR	0.93 (0.76 to 1.14)	0.46	0.9 (0.69 to 1.18)	0.93	Random effect summary estimate was not significant
Shi, et al. 2018	Multidisciplinary care vs. no multidisciplinary care	Any	OR	0.81 (0.61 to 1.08)	0.15	0.9 (0.62 to 1.3)	0.59	Random effect summary estimate was not significant
Valentijn, et al. 2018	Person-centered integrated care vs. control	Any	RR	0.85 (0.7 to 1.04)	0.11	0.86 (0.65 to 1.13)	0.31	Random effect summary estimate was not significant
Silver, et al. 2017	Quality improvement strategy vs. usual care	3-5	RR	0.91 (0.75 to 1.12)	0.37	0.91 (0.68 to 1.22)	0.32	Random effect summary estimate was not significant
Palmer, et al. 2017	Dietary counselling vs. control	3-5	RR	1.58 (0.6 to 4.18)	0.36	1.53 (0.44 to 5.29)	0.72	Random effect summary estimate was not significant
Jun, et al. 2012	Fibrate vs. placebo	3	RR	0.86 (0.63 to 1.19)	0.37	0.8 (0.54 to 1.18)	NA	Random effect summary estimate was not significant
Jun, et al. 2012	Fibrate vs. placebo	1-2	RR	1.02 (0.91 to 1.15)	0.69	1.13 (0.97 to 1.32)	NA	Random effect summary estimate was not significant
Nigwekar, et al. 2016	Folic acid and/or vitamin B6 and/or vitamin B12 vs. control	5D	RR	1 (0.9 to 1.12)	1	1.03 (0.87 to 1.21)	0.11	Random effect summary estimate was not significant
Jardine, et al. 2012	Folic acid and/or vitamin B6 and/or vitamin B12 vs. control	3-5	RR	1.04 (0.93 to 1.16)	0.45	1.02 (0.9 to 1.15)	0.77	Random effect summary estimate was not significant
Hahn, et al. 2018	Low protein diet vs. normal protein diet	3-5ND	RR	0.78 (0.51 to 1.19)	0.25	0.92 (0.54 to 1.56)	0.36	Random effect summary estimate was not significant

* Effect estimate smaller than 1 favors experimental arm (lower mortality in experimental arm); effect estimate larger than 1 favors control arm (lower mortality in control arm)
All statistical tests are two-sided.
Abbreviations: ACEI, angiotensin-converting enzyme inhibitor; ARB, angiotensin receptor blocker; BMS, bare metal stent; CI, confidence interval; CKD, chronic kidney disease; DES, drug-eluting stent; DPP-4, Dipeptidylpeptidase-4; ESA, erythropoiesis-stimulating agent; HD, hemodialysis; HR, hazard ratio; ICD, implantable cardioverter defibrillator; NA, not available; OR, odds ratio; PD, peritoneal dialysis; RR, risk ratio; SGLT-2, sodium glucose cotransporter-2; vs., versus

Table S6. Details of eligible meta-analysis unique in design but ineligible for re-analysis

Author, year	Comparison (experimental arm vs. control arm)	CKD stage	Meta-analysis model	Effect metrics	Effect estimate (95% CI)*	P value	I2 (%)
Koulouridis, et al. 2013	High vs. low first-3-month mean ESA dose in anemia treatment	1-5	Random effects meta-regression analysis	Incidence rate ratio	1.48 (1.02 to 2.14)	NR	NR
Koulouridis, et al. 2013	High vs. low total-study-period mean ESA dose in anemia treatment	1-5	Random effects meta-regression analysis	Incidence rate ratio	1.41 (1.08 to 1.82)	0.01	NR
Shepshelovich, et al. 2016	Intravenous vs. oral iron replacement for anemia treatment	1-5	Random effects meta-analysis	RR	0.94 (0.55 to 1.63)	NR	NR
Charytan, et al. 2016	CABG vs. PCI	3-5ND	Individual patient data meta-analysis	HR	0.99 (0.67 to 1.46)	0.96	NA
Charytan, et al. 2016	CABG vs. PCI	3-5ND	Individual patient data meta-analysis	HR	0.92 (0.54 to 1.58)	NR	NA
Palmer, et al. 2015	ARB vs. placebo	Any with type 2 diabetes	Random effects meta-analysis	OR	0.91 (0.71 to 1.16)	NR	NR
Palmer, et al. 2015	ACEI vs. placebo	Any with type 2 diabetes	Random effects meta-analysis	OR	0.85 (0.61 to 1.19)	NR	NR
Xie, et al. 2016	ACEI vs. placebo	Any	Random effects meta-analysis	OR	0.87 (0.76 to 0.99)	NR	33
Xie, et al. 2016	ARB vs. placebo	Any	Random effects meta-analysis	OR	1.03 (0.89 to 1.21)	NR	0
Xie, et al. 2016	ACEI vs. active control	Any	Random effects meta-analysis	OR	0.69 (0.48 to 0.99)	NR	0
Xie, et al. 2016	ARB vs. active control	Any	Random effects meta-analysis	OR	0.88 (0.71 to 1.1)	NR	0
Xie, et al. 2016	ACEI vs. ARB	Any	Random effects meta-analysis	OR	1.02 (0.36 to 2.91)	NR	NR
Xie, et al. 2016	Combined RAAS blockade vs. single RAAS blockade	Any	Random effects meta-analysis	OR	0.95 (0.77 to 1.16)	NR	46
Major, et al. 2016	Aspirin for preventing cardiovascular diseases vs. placebo	ND	Random effects meta-analysis	RR	0.74 (0.55 to 1)	0.05	0
Wali, et al. 2011	Calvedilol for heart failure vs. placebo	3-5ND	Individual patient data meta-analysis	HR	0.76 (0.63 to 0.93)	0.007	NA
Greeviroj, et al. 2018	Cinacalcet and/or vitamin D analogue or phosphate binders vs. placebo and/or vitamin D analogue or phosphate binders	5D	Random effects meta-analysis	RR	0.97 (0.89 to 1.05)	0.43	0
Das, et al. 2018	Oral patiromer for treating hyperkalemia vs. placebo	3-4 or non-CKD patients with heart failure, with high risk of hyperkalemia	Random effects meta-analysis	RR	0.31 (0.031 to 2.9)	0.3	NR
Herrington, et al. 2016	Statin vs. less statin or placebo, per 1.0 mmol/L reduction in LDL cholesterol	1-2	Individual patient data meta-analysis	RR	0.89 (0.85 to 0.93)	NR	NA
Herrington, et al. 2016	Statin vs. less statin or placebo, per 1.0 mmol/L reduction in LDL cholesterol	3a	Individual patient data meta-analysis	RR	0.92 (0.86 to 0.98)	NR	NA
Herrington, et al. 2016	Statin vs. less statin or placebo, per 1.0 mmol/L reduction in LDL cholesterol	3b	Individual patient data meta-analysis	RR	0.96 (0.88 to 1.04)	NR	NA
Herrington, et al. 2016	Statin vs. less statin or placebo, per 1.0 mmol/L reduction in LDL cholesterol	4-5ND	Individual patient data meta-analysis	RR	0.94 (0.84 to 1.06)	NR	NA
Herrington, et al. 2016	Statin vs. less statin or placebo, per 1.0 mmol/L reduction in LDL cholesterol	5D	Individual patient data meta-analysis	RR	0.97 (0.89 to 1.05)	NR	NA
Peters, et al. 2016	Online hemodiafiltration vs. conventional HD	5	Individual patient data meta-analysis	HR	0.86 (0.75 to 0.99)	NR	NA
He, et al. 2016	Fish oil vs. placebo or other oil	5HD	Fixed effects meta-analysis	RR	0.83 (0.36 to 1.9)	0.66	0

* Effect estimate smaller than 1 favors experimental arm (lower mortality in experimental arm); effect estimate larger than 1 favors control arm (lower mortality in control arm)
All statistical tests are two-sided.
Abbreviations: ACEI, angiotensin-converting enzyme inhibitor; ARB, angiotensin receptor blocker; CABG, coronary artery bypass; CI, confidence interval; CKD, chronic kidney disease; ESA, erythropoiesis-stimulating agent; HD, hemodialysis; HR, hazard ratio; LDL, low-density lipoprotein; NA, not available; NR, not reported; OR, odds ratio; PCI, percutaneous intervention; RAAS, renin–angiotensin–aldosterone system; RR, risk ratio; vs., versus

Table S7. Comparisons of effect of treatment on all-cause mortality between evidences from different chronic kidney disease stages

Comparison (experimental arm vs. control arm)	Study design	Less severe CKD stage				More severe CKD stage				P value for heterogeneity[b]	Statistical significance
		CKD stage	Effect metric	Random effects summary estimate (95% CI)[a]	Deaths / Population	CKD stage	Effect metric	Random effects summary estimate (95% CI)[a]	Deaths / Population		
High vs. low hemoglobin target	RCT	2-5	RR	1.09 (0.99 to 1.2)	1408 / 9951	5D	RR	1.11 (0.95 to 1.3)	483 / 3209	0.85	Both not significant
CABG vs. medical therapy, long-term acm	OS	3-5	RR	0.76 (0.5 to 1.15)	2335 / 6113	5D	RR	0.88 (0.62 to 1.26)	894 / 3160	0.59	Both not significant
CABG vs. medical therapy, short-term acm	OS	3-5	RR	1.06 (0.79 to 1.43)	459 / 3642	5D	RR	1.17 (0.82 to 1.65)	416 / 2645	0.69	Both not significant
CABG vs. PCI, long-term acm	OS	<5	OR	0.82 (0.76 to 0.88)	4327 / 15493	5D	OR	0.92 (0.8 to 1.06)	48664 / 77133	0.13	**Less severe stage**
CABG vs. PCI, short-term acm	OS	3-5	RR	1.81 (1.47 to 2.24)	3470 / 55068	5D	RR	2.28 (1.99 to 2.6)	3347 / 52192	**0.073**	Both significant in same direction
PCI vs. medical therapy, long-term acm	OS	3-5	RR	0.72 (0.6 to 0.86)	3801 / 12647	5D	RR	0.72 (0.52 to 1)	1120 / 3888	1	Both significant in same direction
PCI vs. medical therapy, short-term acm	OS	3-5	RR	0.82 (0.61 to 1.11)	1158 / 7748	5D	RR	0.6 (0.36 to 0.99)	418 / 2854	0.29	**More severe stage**
DES vs. BMS, long-term acm	OS	Any	OR	0.79 (0.71 to 0.89)	>1000 / 117247	5D	OR	0.75 (0.64 to 0.89)	24838 / 62863	0.62	Both significant in same direction
ACEI or ARB vs. control	RCT	Any	OR	0.87 (0.76 to 0.99)	2159 / 17817	5D	RR	0.94 (0.75 to 1.17)	265 / 1746	0.55	**Less severe stage**
More intensive vs. less intensive blood pressure target	RCT	3-5ND	OR	0.86 (0.76 to 0.96)	1293 / 15914	5D	RR	0.8 (0.66 to 0.96)	481 / 1571	0.51	Both significant in same direction
Mineralocorticoid receptor antagonist	RCT	Any	RR	0.58 (0.36 to 0.91)	NR / 1724	5	RR	0.4 (0.23 to 0.7)	59 / 721	0.33	Both significant in same direction
ICD for primary prevention of sudden cardiac death vs. no ICD	OS	3-5	HR	0.74 (0.63 to 0.86)	>1000 / 19808	5D	HR	0.71 (0.54 to 0.92)	NR / 17645	0.81	Both significant in same direction
ICD for primary prevention of sudden cardiac death vs. no ICD	RCT	1	HR	0.48 (0.34 to 0.67)	NR / NR	3b	HR	0.82 (0.66 to 1.01)	NR / NR	**0.0085**	**Less severe stage**
Trans-catheter vs. surgical aortic valve replacement	OS	ND	OR	0.65 (0.41 to 1.03)	792 / 9619	5D	OR	0.78 (0.51 to 1.21)	642 / 8064	0.57	Both not significant
Warfarin for atrial fibrillation vs. control	OS	ND	HR	0.66 (0.6 to 0.72)	>1000 / 30333	5HD	OR	0.91 (0.8 to 1.03)	9088 / 19281	**0.000056**	**Less severe stage**
Cinacalcet vs. control	RCT	3-5	RR	0.97 (0.89 to 1.05)	NR / 8386	5D	RR	0.97 (0.89 to 1.05)	NR / 8632	0.97	Both not significant
Statin vs. less statin or placebo	RCT	ND	RR	0.78 (0.72 to 0.86)	2351 / 33589	5D	RR	0.98 (0.93 to 1.03)	2900 / 7051	**0.000026**	**Less severe stage**
Vitamin D vs. control	RCT	ND	RR	1.55 (0.52 to 4.62)	NR / 832	5D	RR	1.13 (0.63 to 2.03)	NR / 700	0.62	Both not significant
Vitamin D vs. control	OS	ND	RR	0.53 (0.32 to 0.87)	NR / 2729	5D	RR	0.65 (0.57 to 0.75)	>1000 / 218639	0.43	Both significant in same direction
Fibrate vs. placebo	RCT	1-2	RR	1.01 (0.8 to 1.27)	969 / 11408	3	RR	0.86 (0.63 to 1.19)	128 / 918	0.44	Both not significant
Folic acid supplement vs. placebo	RCT	3-5	RR	1.04 (0.93 to 1.16)	756 / 2215	5D	RR	1 (0.9 to 1.12)	819 / 2447	0.6	Both not significant

a. Summary estimate smaller than 1 favors experimental arm (lower mortality in experimental arm); effect estimate larger than 1 favors control arm (lower mortality in control arm)
b. Significance threshold of Cochran's Q test for heterogeneity is p value < 0.1. Significant associations were shown in bold.
All statistical tests are two-sided.
Abbreviations: ACEI, angiotensin-converting enzyme inhibitor; acm, all-cause mortality; ARB, angiotensin receptor blocker; BMS, bare metal stent; CABG, coronary artery bypass; CI, confidence interval; CKD, chronic kidney disease; DES, drug-eluting stent; HR, hazard ratio; ICD, implantable cardioverter defibrillator; NR, not reported; OR, odds ratio; OS, observational study; PCI, percutaneous intervention; RCT, randomized controlled trial; RR, risk ratio; vs., versus

Table S8. Sensitivity subset analysis of prospective studies only of evidence from observational studies graded as convincing or highly suggestive evidence

Author, year	Comparison (experimental arm vs. control arm)	CKD stage	Number of studies	Effect metrics	Summary effect estimate (95% CI) under random effects[a]	Summary estimate p value	I2 (%)	95% prediction interval	Evaluation of bias[b]	Change of level of evidence
Qin, et al. 2016	ACEI or ARB vs. no ACEI or ARB	ND	8	HR	0.83 (0.79 to 0.88)	7E-12	48	0.72 to 0.95	None	Convincing retained
Dahal, et al. 2016	Warfarin for atrial fibrillation vs. no warfarin	ND	2	HR	0.69 (0.62 to 0.77)	1.6E-12	0	NA	Loss of significance under 10% credibility ceiling	Convincing to highly suggestive
Volodarskiy, et al. 2016	CABG vs. PCI	3-5	3	RR	1.15 (0.55 to 2.41)	0.7	48	0 to 1963.73	None	Highly suggestive to no association
Lu, et al. 2017	Vitamin D or analogues vs. non-vitamin D treatment	5D	10	RR	0.56 (0.43 to 0.74)	0.000043	93	0.23 to 1.41	Large heterogeneity; small study effects	Highly suggestive to suggestive
Ravani, et al. 2013	Catheter as HD access vs. fistula	5HD	8	RR	1.34 (1.26 to 1.42)	2.9E-23	15	1.2 to 1.49	Small study effects	Highly suggestive retained
Ravani, et al. 2013	Catheter as HD access vs. graft	5HD	6	RR	1.59 (1.22 to 2.08)	0.00061	90	0.68 to 3.73	Large heterogeneity	Highly suggestive to suggestive
Jin, et al. 2013	Prolonged nocturnal or daytime HD vs. conventional HD	5	6	OR	0.71 (0.6 to 0.85)	0.00017	71	0.42 to 1.2	Large heterogeneity; small study effects	Highly suggestive to suggestive
Smart, et al. 2014	Early referral to specialist nephrology services vs. late referral to specialist nephrology services	Any	4	RR	0.52 (0.29 to 0.94)	0.03	57	0.05 to 5.06	Large heterogeneity; loss of significance under 10% credibility ceiling	Highly suggestive to weak

a. Summary estimate smaller than 1 favors experimental arm (lower mortality in experimental arm); effect estimate larger than 1 favors control arm (lower mortality in control arm)
b. Any of the following: large heterogeneity, signs of small study effects, signs of excess significance bias, and for observational studies, loss of statistical significance in 10% credibility ceiling.
All statistical tests are two-sided.
Abbreviations: ACEI, angiotensin-converting enzyme inhibitor; ARB, angiotensin receptor blocker; CABG, coronary artery bypass grafting; CI, confidence interval; CKD, chronic kidney disease; HD, hemodialysis; HR, hazard ratio; NR, not reported; OR, odds ratio; PCI, percutaneous intervention; RR, risk ratio; vs., versus

Supplementary References

1. Amato L, Addis A, Saulle R, Trotta F, Mitrova Z, Davoli M. Comparative efficacy and safety in ESA biosimilars vs. originators in adults with chronic kidney disease: a systematic review and meta-analysis. J Nephrol. 2018;31(3):321-32.
2. Apetrii M, Goldsmith D, Nistor I, et al. Impact of surgical parathyroidectomy on chronic kidney disease-mineral and bone disorder (CKD-MBD) - A systematic review and meta-analysis. PLoS ONE. 2017;12(11):e0187025.
3. Badve SV, Roberts MA, Hawley CM, et al. Effects of beta-adrenergic antagonists in patients with chronic kidney disease: a systematic review and meta-analysis. J Am Coll Cardiol. 2011;58(11):1152-61.
4. Charytan DM, Desai M, Mathur M, et al. Reduced risk of myocardial infarct and revascularization following coronary artery bypass grafting compared with percutaneous coronary intervention in patients with chronic kidney disease. Kidney Int. 2016;90(2):411-21.
5. Cheng X, Hu Q, Zhao H, Qin S, Zhang D. Transcatheter Versus Surgical Aortic Valve Replacement in Patients With Chronic Kidney Disease: A Meta-Analysis. J Cardiothorac Vasc Anesth. 2018.
6. Crowley MJ, Diamantidis CJ, McDuffie JR, et al. Clinical Outcomes of Metformin Use in Populations With Chronic Kidney Disease, Congestive Heart Failure, or Chronic Liver Disease: A Systematic Review. Ann Intern Med. 2017;166(3):191-200.
7. Dahal K, Kunwar S, Rijal J, Schulman P, Lee J. Stroke, Major Bleeding, and Mortality Outcomes in Warfarin Users With Atrial Fibrillation and Chronic Kidney Disease: A Meta-Analysis of Observational Studies. Chest. 2016;149(4):951-9.
8. Das S, Dey JK, Sen S, Mukherjee R. Efficacy and Safety of Patiromer in Hyperkalemia: A Systematic Review and Meta-Analysis. J Pharm Pract. 2018;31(1):6-17.
9. Fu L, Zhou Q, Zhu W, et al. Do Implantable Cardioverter Defibrillators Reduce Mortality in Patients With Chronic Kidney Disease at All Stages? Int Heart J. 2017;58(3):371-7.
10. Greeviroj P, Kitrungphaiboon T, Katavetin P, et al. Cinacalcet for Treatment of Chronic Kidney Disease-Mineral and Bone Disorder: A Meta-Analysis of Randomized Controlled Trials. Nephron. 2018;139(3):197-210.
11. Habbous S, Przech S, Acedillo R, Sarma S, Garg AX, Martin J. The efficacy and safety of sevelamer and lanthanum versus calcium-containing and iron-based binders in treating hyperphosphatemia in patients with chronic kidney disease: a systematic review and meta-analysis. Nephrol Dial Transplant. 2017;32(1):111-25.
12. Hahn D, Esezobor CI, Elserafy N, Webster AC, Hodson EM. Short-acting erythropoiesis-stimulating agents for anaemia in predialysis patients. Cochrane Database Syst Rev. 2017;1:Cd011690.
13. Hahn D, Hodson EM, Fouque D. Low protein diets for non-diabetic adults with chronic kidney disease. Cochrane Database Syst Rev. 2018;10:Cd001892.
14. Han SS, Park JY, Kang S, et al. Dialysis Modality and Mortality in the Elderly: A Meta-Analysis. Clin J Am Soc Nephrol. 2015;10(6):983-93.
15. He G, Li Q, Li W, Wang L, Yang J, Zeng F. N-Acetylcysteine for Preventing of Acute Kidney Injury in Chronic Kidney Disease Patients Undergoing Cardiac Surgery: A Metaanalysis. Heart Surg Forum. 2018;21(6):E513-e21.
16. He L, Li MS, Lin M, Zhao TY, Gao P. Effect of fish oil supplement in maintenance hemodialysis patients: a systematic review and meta-analysis of published randomized controlled trials. Eur J Clin Pharmacol. 2016;72(2):129-39.
17. Heerspink HJ, Ninomiya T, Zoungas S, et al. Effect of lowering blood pressure on cardiovascular events and mortality in patients on dialysis: a systematic review and meta-analysis of randomised controlled trials. Lancet. 2009;373(9668):1009-15.
18. Herrington WG, Emberson J, Mihaylova B, et al. Impact of renal function on the effects of LDL cholesterol lowering with statin-based regimens: a meta-analysis of individual participant data from 28 randomised trials. Lancet Diabetes Endocrinol. 2016;4(10):829-39.
19. Htay H, Johnson DW, Wiggins KJ, et al. Biocompatible dialysis fluids for peritoneal dialysis. Cochrane Database Syst Rev. 2018;10:Cd007554.
20. Jardine MJ, Kang A, Zoungas S, et al. The effect of folic acid based homocysteine lowering on cardiovascular events in people with kidney disease: systematic review and meta-analysis. BMJ. 2012;344:e3533.
21. Jin HM, Guo LL, Zhan XL, Pan Y. Effect of prolonged weekly hemodialysis on survival of maintenance hemodialysis patients: a meta-analysis of studies. Nephron Clin Pract. 2013;123(3-4):220-8.
22. Jun M, Venkataraman V, Razavian M, et al. Antioxidants for chronic kidney disease. Cochrane Database Syst Rev. 2012;10:Cd008176.
23. Jun M, Zhu B, Tonelli M, et al. Effects of fibrates in kidney disease: a systematic review and meta-analysis. J Am Coll Cardiol. 2012;60(20):2061-71.
24. Kannan A, Poongkunran C, Medina R, Ramanujam V, Poongkunran M, Balamuthusamy S. Coronary Revascularization in Chronic and End-Stage Renal Disease: A Systematic Review and Meta-analysis. Am J Ther. 2016;23(1):e16-28.
25. Kelly JT, Palmer SC, Wai SN, et al. Healthy Dietary Patterns and Risk of Mortality and ESRD in CKD: A Meta-Analysis of Cohort Studies. Clin J Am Soc Nephrol. 2017;12(2):272-9.
26. Khera S, Villablanca PA, Kolte D, et al. Long-Term Outcomes of Drug-Eluting Stents Versus Bare-Metal Stents in End-Stage Renal Disease Patients on Dialysis: A Systematic Review and Meta-Analysis. Cardiol Rev. 2018;26(6):277-86.
27. Koulouridis I, Alfayez M, Trikalinos TA, Balk EM, Jaber BL. Dose of erythropoiesis-stimulating agents and adverse outcomes in CKD: a metaregression analysis. Am J Kidney Dis. 2013;61(1):44-56.
28. Lei H, Yu LT, Wang WN, Zhang SG. Warfarin and the Risk of Death, Stroke, and Major Bleeding in Patients With Atrial Fibrillation Receiving Hemodialysis: A Systematic Review and Meta-Analysis. Front Pharmacol. 2018;9:1218.
29. Li C, Lv L, Wang H, et al. Total parathyroidectomy versus total parathyroidectomy with autotransplantation for secondary hyperparathyroidism: systematic review and meta-analysis. Ren Fail. 2017;39(1):678-87.
30. Li SM, He WB, Chen J, et al. Combined blockade of renin-angiotensin-aldosterone system reduced all-cause but not cardiovascular mortality in dialysis patients: A mediation analysis and systematic review. Atherosclerosis. 2018;269:35-41.
31. Liu Y, Ma X, Zheng J, Jia J, Yan T. Effects of angiotensin-converting enzyme inhibitors and angiotensin receptor blockers on cardiovascular events and residual renal function in dialysis patients: a meta-analysis of randomised controlled trials. BMC Nephrol. 2017;18(1):206.
32. Lo C, Toyama T, Wang Y, et al. Insulin and glucose-lowering agents for treating people with diabetes and chronic kidney disease. Cochrane Database Syst Rev. 2018(9).
33. Lu R, Tang F, Zhang Y, et al. Comparison of Drug-Eluting and Bare Metal Stents in Patients With Chronic Kidney Disease: An Updated Systematic Review and Meta-Analysis. J Am Heart Assoc. 2016;5(11).
34. Lu R, Zhang Y, Zhu X, et al. Effects of mineralocorticoid receptor antagonists on left ventricular mass in chronic kidney disease patients: a systematic review and meta-analysis. Int Urol Nephrol. 2016;48(9):1499-509.
35. Lu RJ, Zhu SM, Tang FL, et al. Effects of vitamin D or its analogues on the mortality of patients with chronic kidney disease: an updated systematic review and meta-analysis. Eur J Clin Nutr. 2017;71(6):683-93.
36. Major RW, Oozeerally I, Dawson S, Riddleston H, Gray LJ, Brunskill NJ. Aspirin and cardiovascular primary prevention in non-endstage chronic kidney disease: A meta-analysis. Atherosclerosis. 2016;251:177-82.
37. Malhotra R, Nguyen HA, Benavente O, et al. Association Between More Intensive vs Less Intensive Blood Pressure Lowering and Risk of Mortality in Chronic Kidney Disease Stages 3 to 5: A Systematic Review and Meta-analysis. JAMA Intern Med. 2017;177(10):1498-505.
38. Mathew A, McLeggon JA, Mehta N, et al. Mortality and Hospitalizations in Intensive Dialysis: A Systematic Review and Meta-Analysis. Can J Kidney Health Dis. 2018;5:2054358117749531.
39. McCann M, Moore ZE. Interventions for preventing infectious complications in haemodialysis patients with central venous catheters. Cochrane Database Syst Rev. 2010(1):Cd006894.
40. Nigwekar SU, Kang A, Zoungas S, et al. Interventions for lowering plasma homocysteine levels in dialysis patients. Cochrane Database Syst Rev. 2016(5):Cd004683.
41. Nistor I, De Sutter J, Drechsler C, et al. Effect of renin-angiotensin-aldosterone system blockade in adults with diabetes mellitus and advanced chronic kidney disease not on dialysis: a systematic review and meta-analysis. Nephrol Dial Transplant. 2018;33(1):12-22.
42. Nistor I, Palmer SC, Craig JC, et al. Haemodiafiltration, haemofiltration and haemodialysis for end-stage kidney disease. Cochrane Database Syst Rev. 2015(5):Cd006258.
43. Palmer SC, Di Micco L, Razavian M, et al. Antiplatelet agents for chronic kidney disease. Cochrane Database Syst Rev. 2013(2):Cd008834.
44. Palmer SC, Maggo JK, Campbell KL, et al. Dietary interventions for adults with chronic kidney disease. Cochrane Database Syst Rev. 2017;4:Cd011998.
45. Palmer SC, Mavridis D, Navarese E, et al. Comparative efficacy and safety of blood pressure-lowering agents in adults with diabetes and kidney disease: a network meta-analysis. Lancet. 2015;385(9982):2047-56.
46. Palmer SC, Navaneethan SD, Craig JC, et al. Meta-analysis: erythropoiesis-stimulating agents in patients with chronic kidney disease. Ann Intern Med. 2010;153(1):23-33.
47. Palmer SC, Saglimbene V, Craig JC, Navaneethan SD, Strippoli GF. Darbepoetin for the anaemia of chronic kidney disease. Cochrane Database Syst Rev. 2014(3):Cd009297.
48. Palmer SC, Saglimbene V, Mavridis D, et al. Erythropoiesis-stimulating agents for anaemia in adults with chronic kidney disease: a network meta-analysis. Cochrane Database Syst Rev. 2014(12):Cd010590.
49. Peters SA, Bots ML, Canaud B, et al. Haemodiafiltration and mortality in end-stage kidney disease patients: a pooled individual participant data analysis from four randomized controlled trials. Nephrol Dial Transplant. 2016;31(6):978-84.
50. Phan K, Zhao DF, Zhou JJ, Karagaratnam A, Phan S, Yan TD. Bioprosthetic versus mechanical prostheses for valve replacement in end-stage renal disease patients: systematic review and meta-analysis. J Thorac Dis. 2016;8(5):769-77.
51. Pun PH, Al-Khatib SM, Han JY, et al. Implantable cardioverter-defibrillators for primary prevention of sudden cardiac death in CKD: a meta-analysis of patient-level data from 3 randomized trials. Am J Kidney Dis. 2014;64(1):32-9.

52. Qin Y, Chen T, Chen Q, et al. The effect of angiotensin-converting enzyme inhibitor/angiotensin receptor blocker use on mortality in patients with chronic kidney disease: a meta-analysis of observational studies. Pharmacoepidemiol Drug Saf. 2016;25(5):503-11.
53. Quach K, Lvtvyn L, Baigent C, et al. The Safety and Efficacy of Mineralocorticoid Receptor Antagonists in Patients Who Require Dialysis: A Systematic Review and Meta-analysis. Am J Kidney Dis. 2016;68(4):591-8.
54. Ravani P, Palmer SC, Oliver MJ, et al. Associations between hemodialysis access type and clinical outcomes: a systematic review. J Am Soc Nephrol. 2013;24(3):465-73.
55. Remschmidt C, Wichmann O, Harder T. Influenza vaccination in patients with end-stage renal disease: systematic review and assessment of quality of evidence related to vaccine efficacy, effectiveness, and safety. BMC Med. 2014;12:244.
56. Ren X, Liu W, Peng Y, et al. Percutaneous coronary intervention compared with coronary artery bypass graft in coronary artery disease patients with chronic kidney disease: a systematic review and meta-analysis. Ren Fail. 2014;36(8):1177-86.
57. Ruospo M, Palmer SC, Natale P, et al. Phosphate binders for preventing and treating chronic kidney disease-mineral and bone disorder (CKD-MBD). Cochrane Database Syst Rev. 2018;8:Cd006023.
58. Sampson AL, Singer RF, Walters GD. Uric acid lowering therapies for preventing or delaying the progression of chronic kidney disease. Cochrane Database Syst Rev. 2017;10:Cd009460.
59. Scotland G, Cruickshank M, Jacobsen E, et al. Multiple-frequency bioimpedance devices for fluid management in people with chronic kidney disease receiving dialysis: a systematic review and economic evaluation. Health Technol Assess. 2018;22(1):1-138.
60. Sekercioglu N, Thabane L, Diaz Martinez JP, et al. Comparative Effectiveness of Phosphate Binders in Patients with Chronic Kidney Disease: A Systematic Review and Network Meta-Analysis. PLoS ONE. 2016;11(6):e0156891.
61. Sharma P, Blackburn RC, Parke CL, McCullough K, Marks A, Black C. Angiotensin-converting enzyme inhibitors and angiotensin receptor blockers for adults with early (stage 1 to 3) non-diabetic chronic kidney disease. Cochrane Database Syst Rev. 2011(10):Cd007751.
62. Shaw C, Nitsch D, Lee J, Fogarty D, Sharpe CC. Impact of an Early Invasive Strategy versus Conservative Strategy for Unstable Angina and Non-ST Elevation Acute Coronary Syndrome in Patients with Chronic Kidney Disease: A Systematic Review. PLoS ONE. 2016;11(5):e0153478.
63. Shepshelovich D, Rozen-Zvi B, Avni T, Gafter U, Gafter-Gvili A. Intravenous Versus Oral Iron Supplementation for the Treatment of Anemia in CKD: An Updated Systematic Review and Meta-analysis. Am J Kidney Dis. 2016;68(5):677-90.
64. Shi Y, Xiong J, Chen Y, et al. The effectiveness of multidisciplinary care models for patients with chronic kidney disease: a systematic review and meta-analysis. Int Urol Nephrol. 2018;50(2):301-12.
65. Shurrab M, Ko DT, Zayed Y, et al. Outcomes of ICDs and CRTs in patients with chronic kidney disease: a meta-analysis of 21,000 patients. J Interv Card Electrophysiol. 2018;53(1):123-9.
66. Silver SA, Bell CM, Chertow GM, et al. Effectiveness of Quality Improvement Strategies for the Management of CKD: A Meta-Analysis. Clin J Am Soc Nephrol. 2017;12(10):1601-14.
67. Smart NA, Dieberg G, Ladhani M, Titus T. Early referral to specialist nephrology services for preventing the progression to end-stage kidney disease. Cochrane Database Syst Rev. 2014(6):Cd007333.
68. Song K, Jiang S, Shi Y, Shen H, Shi X, Jing D. Renal replacement therapy for prevention of contrast-induced acute kidney injury: a meta-analysis of randomized controlled trials. Am J Nephrol. 2010;32(5):497-504.
69. Sun L, Zou L, Chen M, Liu B. Meta-analysis of statin therapy in maintenance dialysis patients. Ren Fail. 2015;37(7):1149-56.
70. Tan W, Wang Q, Xin G, et al. Different hemodialysis methods for survival of patients with maintenance hemodialysis: A meta-analysis. Int J Clin Exp Med. 2018;11(5):4389-96.
71. Toyama T, Neuen BL, Jun M, et al. Effect of SGLT2 inhibitors on cardiovascular, renal and safety outcomes in patients with type 2 diabetes mellitus and chronic kidney disease: A systematic review and meta-analysis. Diabetes Obes Metab. 2019.
72. Valentijn PP, Pereira FA, Ruospo M, et al. Person-Centered Integrated Care for Chronic Kidney Disease: A Systematic Review and Meta-Analysis of Randomized Controlled Trials. Clin J Am Soc Nephrol. 2018;13(3):375-86.
73. Volodarskiy A, Kumar S, Amin S, Bangalore S. Optimal Treatment Strategies in Patients with Chronic Kidney Disease and Coronary Artery Disease. Am J Med. 2016;129(12):1288-98.
74. Volodarskiy A, Kumar S, Pracon R, et al. Drug-Eluting vs Bare-Metal Stents in Patients With Chronic Kidney Disease and Coronary Artery Disease: Insights From a Systematic Review and Meta-Analysis. J Invasive Cardiol. 2018;30(1):10-7.
75. Wali RK, Iyengar M, Beck GJ, et al. Efficacy and safety of carvedilol in treatment of heart failure with chronic kidney disease: a meta-analysis of randomized trials. Circ Heart Fail. 2011;4(1):18-26.
76. Wang AY, Ninomiya T, Al-Kahwa A, et al. Effect of hemodiafiltration or hemofiltration compared with hemodialysis on mortality and cardiovascular disease in chronic kidney failure: a systematic review and meta-analysis of randomized trials. Am J Kidney Dis. 2014;63(6):968-78.
77. Wang F, Lu X, Zhang J, Xiong R, Li H, Wang S. Effect of Lanthanum Carbonate on All-Cause Mortality in Patients Receiving Maintenance Hemodialysis: a Meta-Analysis of Randomized Controlled Trials. Kidney Blood Press Res. 2018;43(2):536-44.
78. Wang G, Liu H, Wang C, Ji X, Gu W, Mu Y. Cinacalcet versus Placebo for secondary hyperparathyroidism in chronic kidney disease patients: a meta-analysis of randomized controlled trials and trial sequential analysis. Sci Rep. 2018;8(1):3111.
79. Wang Y, Ivany JN, Perkovic V, Gallagher MP, Woodward M, Jardine MJ. Anticoagulants and antiplatelet agents for preventing central venous haemodialysis catheter malfunction in patients with end-stage kidney disease. Cochrane Database Syst Rev. 2016;4:Cd009631.
80. Wang Y, Zhu S, Gao P, Chen Y, Zhang Q. Drug-Eluting Stents Versus Bare-Metal Stents in Patients With End-Stage Renal Disease. Am J Med Sci. 2018;355(4):331-41.
81. Wang Y, Zhu S, Gao P, Zhou J, Zhang Q. Off-pump versus on-pump coronary surgery in patients with chronic kidney disease: a meta-analysis. Clin Exp Nephrol. 2018;22(1):99-109.
82. Wongrakpanich S, Susantitaphong P, Isaranuwatchai S, Chenbhanich J, Eiam-Ong S, Jaber BL. Dialysis Therapy and Conservative Management of Advanced Chronic Kidney Disease in the Elderly: A Systematic Review. Nephron. 2017;137(3):178-89.
83. Xie J, Kiryluk K, Ren H, et al. Coiled versus straight peritoneal dialysis catheters: a randomized controlled trial and meta-analysis. Am J Kidney Dis. 2011;58(6):946-55.
84. Xie X, Liu Y, Perkovic V, et al. Renin-Angiotensin System Inhibitors and Kidney and Cardiovascular Outcomes in Patients With CKD: A Bayesian Network Meta-analysis of Randomized Clinical Trials. Am J Kidney Dis. 2016;67(5):728-41.
85. Yang M, Xie XS, Yuan WJ. A meta-analysis of the effects of statin treatment on cardiovascular events and all-cause mortality in diabetic dialysis patients. Int J Clin Exp Med. 2015;8(6):8415-24.
86. Yang X, Liu Q, Yuan L, Wang H, Xie Q. Comparison of Limus-Eluting and Paclitaxel-Eluting Stents for Coronary Intervention in Patients with Chronic Kidney Disease. Int Heart J. 2018;59(6):1202-10.
87. Ye Y, Liu H, Chen Y, et al. Hemoglobin targets for the anemia in patients with dialysis-dependent chronic kidney disease: a meta-analysis of randomized, controlled trials. Ren Fail. 2018;40(1):671-9.
88. Zeng X, Lincoff AM, Schulz-Schupke S, et al. Efficacy and safety of bivalirudin in coronary artery disease patients with mild to moderate chronic kidney disease: Meta-analysis. J Cardiol. 2018;71(5):494-504.
89. Zhang X, Xiang C, Zhou YH, Jiang A, Qin YY, He J. Effect of statins on cardiovascular events in patients with mild to moderate chronic kidney disease: a systematic review and meta-analysis of randomized clinical trials. BMC Cardiovasc Disord. 2014;14:19.
90. Zhao HJ, Li Y, Liu SM, et al. Effect of calcium channels blockers and inhibitors of the renin-angiotensin system on renal outcomes and mortality in patients suffering from chronic kidney disease: systematic review and meta-analysis. Ren Fail. 2016;38(6):849-56.
91. Zhao Y, Pei X, Zhao W. Timing of Dialysis Initiation and Mortality Risk in Chronic Kidney Disease: A Meta-Analysis. Ther Apher Dial. 2018;22(6):600-8.
92. Zhou C, Gu Y, Mei C, Dai B, Wang Y, Xue C. Dialysis modality and mortality in polycystic kidney disease. Hemodial Int. 2018;22(4):515-23.
